# The parisite–(Ce) enigma: challenges in the identification of fluorcarbonate minerals

**DOI:** 10.1007/s00710-020-00723-x

**Published:** 2020-10-10

**Authors:** Manuela Zeug, Lutz Nasdala, Martin Ende, Gerlinde Habler, Christoph Hauzenberger, Chutimun Chanmuang N., Radek Škoda, Dan Topa, Manfred Wildner, Richard Wirth

**Affiliations:** 1 Institut für Mineralogie und Kristallographie, Universität Wien, Althanstr. 14, 1090 Wien, Austria; 2 Department of Lithospheric Research, University of Vienna, Althanstr. 14, 1090 Wien, Austria; 3 NAWI Graz Geozentrum, Universitätsplatz 2, 8010, Graz, Austria; 4 Department of Geological Sciences, Masaryk University, Kotlářská 2, 61137 Brno, Czech Republic; 5 Natural History Museum Vienna, Burgring 7, 1010 Wien, Austria; 6 Helmholtz-Zentrum Potsdam – GFZ German Research Centre for Geosciences, Telegrafenberg, 14473 Potsdam, Germany

**Keywords:** Parisite–(Ce), Röntgenite–(Ce), Fluorcarbonate, Polycrystal, Raman spectroscopy, Stacking pattern

## Abstract

A multi-methodological study was conducted in order to provide further insight into the structural and compositional complexity of rare earth element (REE) fluorcarbonates, with particular attention to their correct assignment to a mineral species. Polycrystals from La Pita Mine, Municipality de Maripí, Boyacá Department, Colombia, show syntaxic intergrowth of parisite–(Ce) with röntgenite–(Ce) and a phase which is assigned to *B*_*3*_*S*_*4*_ (i.e., bastnäsite-3–synchisite-4; still unnamed) fluorcarbonate. Transmission electron microscope (TEM) images reveal well-ordered stacking patterns of two monoclinic polytypes of parisite–(Ce) as well as heavily disordered layer sequences with varying lattice fringe spacings. The crystal structure refinement from single crystal X-ray diffraction data – impeded by twinning, complex stacking patterns, sequential and compositional faults – indicates that the dominant parisite–(Ce) polytype *M*_1_ has space group *Cc*. Parisite–(Ce), the *B*_*3*_*S*_*4*_ phase and röntgenite–(Ce) show different BSE intensities from high to low. Raman spectroscopic analyses of parisite–(Ce), the *B*_*3*_*S*_*4*_ phase and röntgenite–(Ce) reveal different intensity ratios of the three symmetric CO_3_ stretching bands at around 1100 cm^−1^. We propose to non-destructively differentiate parisite–(Ce) and röntgenite–(Ce) by their 1092 cm^−1^ / 1081 cm^−1^ ν_1_(CO_3_) band height ratio.

**Electronic supplementary material:**

The online version of this article (10.1007/s00710-020-00723-x) contains supplementary material, which is available to authorized users.

## Introduction

Parisite–(Ce), idealised formula CaCe_2_(CO_3_)_3_F_2_, belongs to the group of REE fluorcarbonate minerals. The study of fluorcarbonate minerals has increased appreciably because the majority of REEs worldwide is contained in these minerals (Williams-Jones and Wood 1992; Smith et al. 1999; Castor 2008; Gysi and Williams-Jones 2015). For instance, extensive research addressing the thermodynamic stability of fluorcarbonates has been conducted by Williams-Jones and Wood (1992) and Gysi and Williams-Jones (2015), in order to provide information on REE ore formation.

Bastnäsite, REECO_3_F, and synchysite, CaREE(CO_3_)_2_F, represent end members of a polysomatic mineral series including parisite, CaREE_2_(CO_3_)_3_F_2_, and röntgenite, Ca_2_REE_3_(CO_3_)_5_F_3_, as intermediate members. All of these minerals are characterised by a layered topology that is composed of bastnäsite (*B*) and synchisite (*S*) basic units (e.g. Capitani 2019). They have occasionally been considered to form one single “bastnäsite-synchisite series” (e.g. Van Landuyt and Amelinckx 1975). This consideration appeared practical as the above REE fluorcarbonates are characterised by complicated mixed-layer structures consisting of complex syntaxic intergrowths of virtually all members except of bastnäsite–synchysite intergrowths (Donnay and Donnay 1953). The majority of REE fluorcarbonates are hence polycrystals. This raises the question, whether or not a specific mineral name can be used for a “crystal” consisting of polysomatic layering sequences? The fluorine is commonly substituted by an OH^−^ group and the extent of this substitution varies from negligible to predominance of OH^−^ over F.

The first description of “parisite” appeared in the middle of the nineteenth century: The Italian mineralogist Lavinio de Medici-Spada used this term in describing a specimen found in the Muzo emerald-mining area, Boyacá Department, Colombia (Bunsen 1854). “Parisite” has been named after the former mine owner and manager, Mr. José J. Paris. Only after 1890, further occurrences were discovered. Parisite–(Ce), which is one of the most common fluorcarbonate species, is known from the carbonatite orebody of Mountain Pass, California (Castor 2008), the carbonatite complex Amba Dongar, India, (Doroshkevich et al. 2009), the alkaline granite-syenite pegmatites of the Mount Malosa pluton in Malawi (Guastoni et al. 2009, 2010), the pegmatitic carbonatite of the Snowbird mine, Montana, (Metz et al. 1985), the ultramafic lamprophyre–carbonatite complex near Delitzsch, Germany (Seifert et al. 2000), and the Bayan Obo deposit, Inner Mongolia, China (Smith et al. 1999). The much rarer mineral parisite–(Nd) was described to occur in the Bayan Obo deposit, China (Zhang and Tao 1986), and parisite–(La) in the Mula mine, Novo Horizonte, Bahia, Brazil (Menezes Filho et al. 2018).

**Fig. 1:**
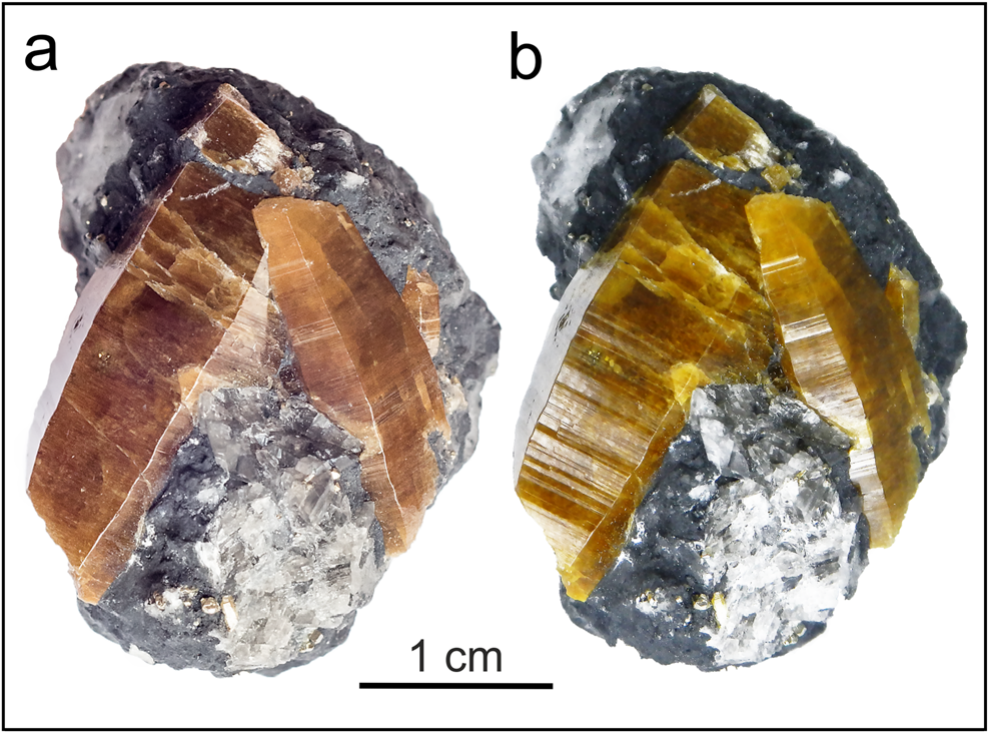
Parisite–(Ce) specimen from the La Pita Mine, Maripí, Colombia. **a** Image obtained in daylight. **b** Image obtained under artificial illumination. Note the colour change from reddish brown to yellowish brown. Striation of main prism faces perpendicular to the prism direction is clearly seen in the right image

The use of parisite–(Ce), as gemstone is rather unusual, especially for samples from the Colombian emerald deposits. These specimens are rarely transparent and flawless without inclusions or impurities. Moreover, parisite–(Ce) is not easy to handle for gem cutters as specimens are decidedly brittle and fractured and have low hardness (~4.5 on the Mohs hardness scale). Nevertheless, parisite–(Ce) is quite well represented in the Colombian gem trade, presumably owing to its attractive colour change between daylight and artificial illumination (Fig. 1). Here, we present the results of a comprehensive chemical and structural characterisation of parisite–(Ce) from La Pita mine, Muzo area, Colombia. Our study aimed at resolving stacking patterns within mixed-layer compounds, thus providing further insight into the syntaxic intergrowth of REE fluorcarbonates. Furthermore, our study aimed at providing a spectroscopy-based in-situ identification of REE fluorcarbonates, in particular of the spectroscopically similar species parisite–(Ce) and röntgenite–(Ce).

## Background information

### Geological setting and formation conditions

The Cordilliera Mountains in Colombia are divided into three ranges, namely, the westernmost Cordillera Occidental, the Cordillera Central and the easternmost Cordillera Oriental. The Cordillera Oriental hosts two main mining districts, Muzo–Coscuez in the northwest and Chivor–Gachalá in the southeast (e.g. Bosshart 1991). The mineralisations are widely similar in the western and eastern emerald mining areas. Minor differences include the formation ages [western zone 38–32 Ma (Branquet et al. 1999); eastern zone ~65 Ma (Cheilletz et al. 1997)] and how mineralising fluids were formed (allochthonous in the western zone and autochthonous in the eastern zone).

Parisite–(Ce) crystals investigated in the present study originated from La Pita mine, Municipality de Maripí, Boyacá Department, Colombia, which is situated in the western belt of the Eastern Cordilliera or Cordillera Oriental (Cheilletz et al. 1994). According to Ringsrud and Boehm (2013), the Muzo mining region comprises the Muto, Peñas Blancas, Muzo and Coscuez mines. Usually parisite–(Ce) forms in alkaline igneous rocks such as sodic granite, syenite, trachyte or carbonatite. Colombian parisite–(Ce) represents an exception, as it is found in veinlets and pockets within carbonaceous sediments (Cook 2000). In the La Pita mine, the mineral paragenesis containing parisite–(Ce) and the famous Colombian emerald occurs in 120–130 Ma old organic rich blackshales (e.g. Bosshart 1991; Ottaway et al. 1994).

The western zone of Cordillera Oriental developed during a compressive tectonic phase (Laumonier et al. 1996). Cheilletz and Giuliani (1996) have elaborated two formation stages of extensional vein systems. In the first stage, veins with fibrous calcite and pyrite were generated and hydrothermal fluids led to formation of albite and calcite. During this phase, thermal reduction of evaporitic sulphur led to sulphur reaction with the organic rich blackshales and consequently to oxidation of organic matter, which then released organically bounded major elements (Si, Al, K, Ti, Mg, P), trace elements (Ba, Be, Cr, V,C, B, U), and REEs (Ottaway et al. 1994; Cheilletz and Giuliani 1996). In the second stage, overpressured fluids infiltrated fractures, which led to remineralisation of calcite, dolomite, pyrite and muscovite in extensional veins and hydraulic breccias, and precipitation of fluorite, apatite, parisite–(Ce), REE bearing dolomite, emerald and quartz in cavities (Cheilletz and Giuliani 1996). In the emerald mining area, parisite–(Ce) and fluorite are used as indicators for emerald mineralisation (Ottaway et al. 1994). The trapping temperatures of coexisting minerals from stage 2, such as albite, muscovite or emerald, were around 300 °C (Cheilletz et al. 1994; Ottaway et al. 1994; Giuliani et al. 1995), whereas the trapping temperature of quartz has been estimated around 270 °C (Dubois 1994). Parisite–(Ce) has been found as inclusions in Colombian emerald (see page 252 in Gübelin and Koivula 1986) and quartz (Muyal 2015). These observations suggest a syngenetic origin of parisite–(Ce), emerald and quartz. Furthermore, they support the assumption for a low-temperature genesis of emerald and quartz, inasmuch as parisite–(Ce) decomposes irreversibly into REE oxyfluorides, CaCO_3_ and CO_2_ at around 350 °C (664 K, Gysi and Williams-Jones 2015).

### Crystallography of fluorcarbonates

The determination of the crystal structure of parisite–(Ce) and other REE fluorcarbonates is complex. This is due to polysomatic and/or polytypic stacking sequences, consisting of fluorcarbonate phases, parallel to (0 0 1) of the hexagonal or pseudohexagonal (monoclinic) unit cell. In the present study, the term “polysomatic” is referred to compositional disorder, while the term “polytypic” describes structural order/disorder of layers that have the same composition (cf. Capitani 2019). Instead of “polytypic” and “polysomatic”, the terms “sequential” and “compositional” may be used, respectively (cf. van Landuyt and Amelinckx 1975). Both terms are used to describe the complicated more or less periodic changes of layer series in fluorcarbonate minerals. Changes in phase composition are caused by compositional faults, which may lead to a distinct polysome provided compositional faults occur periodically. In contrast, a sequential fault defines layer type changes without affecting the composition, which may lead to a new polytype (van Landuyt and Amelinckx 1975; Capitani 2019). In general, polysomatic faults are easier to reveal than polytypic faults, because compositional changes are clearly visible from lattice fringe spacing, whereas structural faults only affect the orientation of layers and therefore are easily overlooked (Capitani 2019).

Due to the present lack of unequivocal nomenclature, the description of the complex stacking-layer series in parisite–(Ce) and other fluorcarbonates is inconsistent within the literature. The existing nomenclatures are briefly reviewed here. Donnay and Donnay (1953) have introduced the terms “*B*” slab and “*S*” slab to describe the layering sequences of REE fluorcarbonates, where *B* stands for the Ca free endmember bastnäsite–(Ce), and *S* for synchisite–(Ce). Furthermore, Donnay and Donnay (1953) suggested the following terms for layers in fluorcarbonates: REE-F-layer (*d*), CO_3_ layer between two REE-F-layers (*e*), Ca-layer (*f*), CO_3_-layer between Ca- and REE-F-layer (*g*). As a result, parisite–(Ce) has the ideal stacking sequence *de* (*B*, bastnäsite-layer) and *dgfg* (*S*, synchysite-layer) and the ideal stacking sequence of röntgenite–(Ce) is de (*B*) and 2 × {*dgfg*} (*S*_2_). Van Landuyt and Amelinckx (1975) proposed to use *BmSn* for the characterisation of layer sequences in fluorcarbonate minerals, where the suffix *m* and *n* quote the number of *B* and *S* slabs in a sequence. The latter nomenclature is nowadays most commonly used in the literature. However, neither the *dgfg* code nor the *BmSn* notation seemed practicable for describing TEM observations. The first code (*dgfg*) failed to be concise whereas the second notation (*BmSn*) is too concise to describe layers within one polysome. Therefore, Capitani (2019) developed a new notation, which has proven quite useful for the interpretation of high-resolution TEM (HR–TEM) images. Capitani (2019) introduced a notation with V for CaCO_3_ layers and *B* for bastnäsite [Ce(CO_3_)F] layers, because these can be easily identified in HR–TEM images. In TEM images obtained along [1 $\overset{\hbox{--} }{1}$ 0] the *V*-layer appears as a wide grey band (*f*- or Ca-layer) enclosed by two white dotted lines (*g*- or CO_3_-layers), the *B*-layer is composed of two dark lines (*d*- or REE-F-layer) separated by a thin bright dotted line (*e*- or CO_3_-layer). A comprehensive translation of the different codings of fluorcarbonate phases is given by Capitani (2019). It should be mentioned that another advanced, fairly complex notation was introduced by Yang et al. (1998). The latter, however, is not considered in the present study, because it is even more impracticably detailed than the *defg*-notation of Donnay and Donnay (1953).

In most cases, parisite–(Ce) occurs in the form of polycrystals, which is due to the syntaxic intergrowth of at least two species (Donnay and Donnay 1953). The term “syntaxy” was introduced by Ungemach (1935) to describe the oriented intergrowth of two species having the same chemical composition, hence considering syntaxic intergrowth as a special case of epitaxic intergrowth and it is listed as nomenclature recommendation in the “Report of the International Mineralogical Association (IMA) - International Union of Crystallography (IUCr) Joint Committee on Nomenclature” (Bailey 1977). For many years, unravelling the crystal structure of parisite–(Ce) was impossible because of this mineral’s complex polytypic disorder. Both hexagonal symmetry with the space group *R*3 (Donnay and Donnay 1953) and monoclinic symmetry with *m* or 2 *m* symmetry (Ni et al. 2000) were determined in the past. Reduction in symmetry from hexagonal to monoclinic in parisite–(Ce) and synchisite–(Ce) is caused by insertion of Ca-layers in the structure (Ni et al. 2000). The assignment to space group *R*3 by Donnay and Donnay (1953) was based only on the symmetry of heavy atoms and whereas it did not consider the CO-layer stacking; this assignment is therefore potentially incorrect.

A further argument for a monoclinic symmetry of parisite–(Ce) can be found in the study of Capitani (2019). This author has compared his observations from parisite–(Ce) from Mount Malosa (Malawi) with observations from parisite–(Ce) from occurrences in China (Wu et al. 1998; Meng et al. 2001a, 2001b, 2002) and Olympic Dam deposit, Australia (Kontonikas-Charos et al. 2017). Long-range polysomes were identified from the Chinese samples, whereas short-range stacking disorder and periodic bastnäsite–parisite repetitions were identified from the Australian samples, consistent with observations made by Capitani (2019). Ciobanu et al. (2017) referred the variable characteristics to differences in growth rates (long range stacking disorder is supposed to develop at slow growth rates, whereas short range stacking order is linked to fast growth rates) and concluded that the growth rate affects the Ca–CO_3_ arrangement, which leads to monoclinic symmetry in the former and hexagonal/rhombohedral symmetry in the latter case. These observations could not be supported by the results of Capitani (2019), because although the data for Mount Malosa fluorcarbonates belong to the short stacking disorder, they show monoclinic symmetry. Capitani (2019) also stated that the HAADF imaging method used by Ciobanu et al. (2017) is unable to reveal the actual symmetry of Ca,REE fluorcarbonates because this technique – in contrast to HR–TEM imaging – is not sensitive to light elements such as C and O. Only HR–TEM can provide insight into the different stacking arrangements of the CO_3_ layers, which reveal the monoclinic symmetry as well as the polytypic disorder.

## Samples and experimental

### Samples and preparation

The present investigation was carried out on four parisite–(Ce) crystals from the La Pita mine, Municipality de Maripí, Boyacá Department, Colombia. All samples show brown to reddish brown colour in daylight and a rather yellowish brown in artificial light (Fig. 1). Crystal #3 is macroscopically transparent and virtually free of inclusions, whereas crystals #1, #2 and #4 are semi- to non-transparent and rich in inclusions. Crystals are mostly prismatic to barrel-shaped in appearance; they are dominated by hexagonal dipyramid faces that are striated perpendicular to the ***c*** axis, and basal pinacoid faces.

**Fig. 2:**
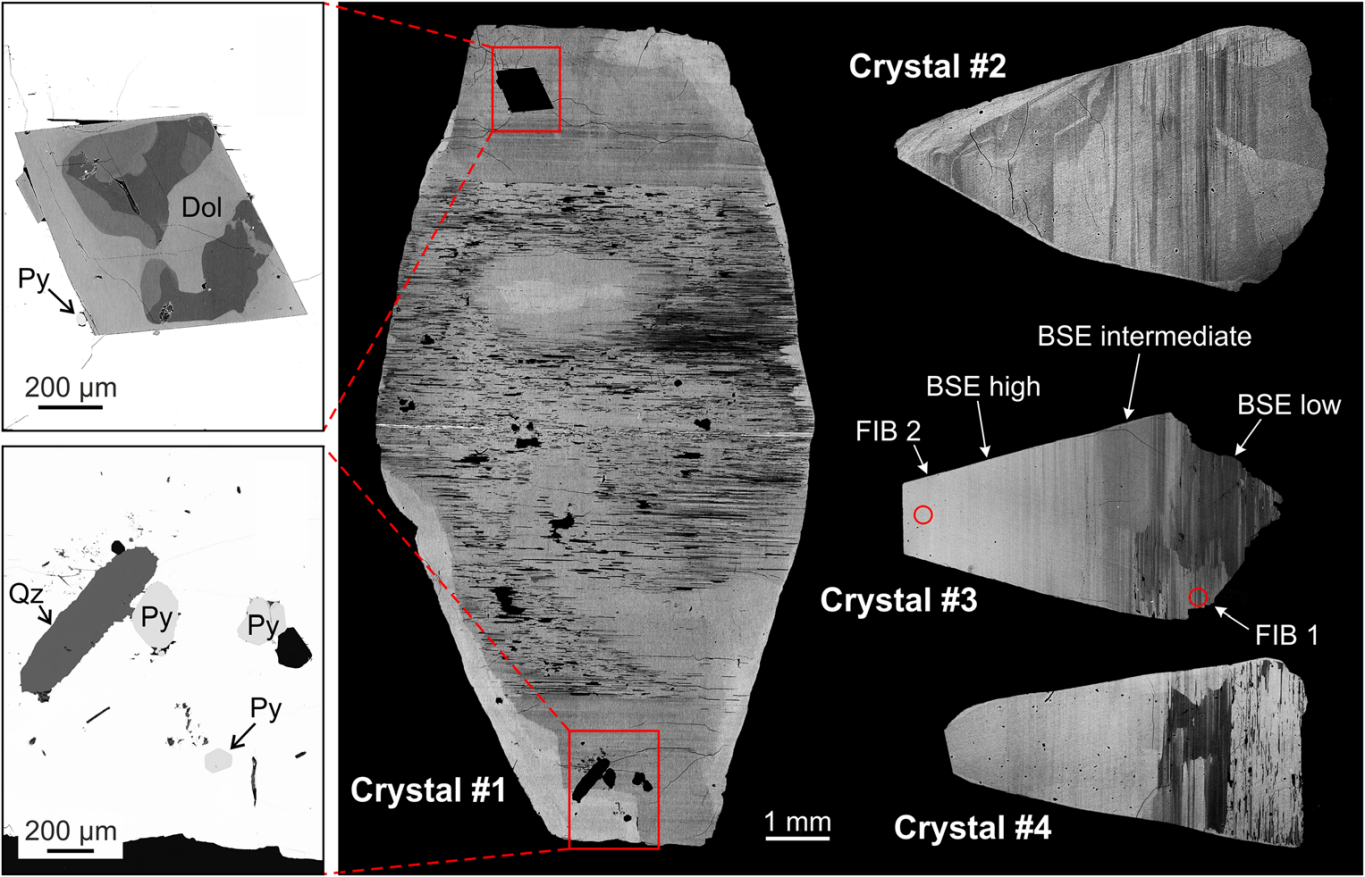
BSE images of the four parisite–(Ce) crystals investigated. Contrast and brightness were adjusted individually for each BSE image and hence cannot be directly compared among images. All crystals show a distinct layering structure perpendicular to the c axes. Crystal #1 contains mm-sized inclusions of dolomite (Dol), pyrite (Py), and quartz (Qz; abbreviations according to Whitney and Evans 2010). Note that BSE intensities of the inclusions are much lower, compared to that of the host parisite–(Ce) crystal. Internal BSE intensity variations of the dolomite crystal are due to variations in the chemical composition (BSE decreases with increasing Mg and decreasing Fe and Ca contents). BSE intensity variations of crystal #3 indicate the presence of three interior regions (marked with arrows). Red circles mark the locations where two TEM foils were extracted

Prior to sample preparation, mass density values were determined by weighing crystals in water and in air. Assuming a monoclinic symmetry, crystals were oriented before cutting using a Nonius Kappa four-circle, single-crystal X-ray diffractometer equipped with a charge-coupled device (CCD) area detector. Specimens were then cut in half using a diamond-coated steel wire. Crystals #1 and #3 were cut along the *a*–*c* plane, crystal #2 was cut along the *b*–*c* plane, and crystal #4 was cut with random orientation along the ***c*** axis. At first a few slices were separated from crystal #4 (~635 μm thickness) for optical absorption spectroscopy. One half of each crystal was embedded in epoxy resin and ground and polished. Small chips for single crystal X–ray diffraction were cut out of crystal #3. For TEM analysis, two thin foils of rectangular shape (ca. 18 μm × 11 μm) were extracted from crystal #3 by Focused Ion Beam (FIB) preparation. One foil was extracted from an area showing strong heterogeneity in BSE signal intensity, whereas the other stems from an apparently homogeneous region (Fig. 2). Crystal #3 was embedded with the *a–c* plane plane-parallel to the surface. Both foils were extracted perpendicular to the surface assuming an orientation parallel (1 0 0). Focused ion-beam preparation was done using a FEI Quanta 3D FEG dual beam scanning electron microscope (SEM) equipped with a field-emission Ga liquid-metal ion source, Pt and C gas-injection systems, and an Omniprobe 100.7 micromanipulator. The accelerating voltage was set to 30 kV throughout the sputtering and gas deposition procedure. During foil preparation, the ion beam current was successively reduced from 65 to 1 nA for foil extraction, 500–300 pA for thinning, and 100 pA for final surface cleaning. Platinum deposition was used for sample surface protection, prevention of selective milling, mechanical stabilization of the foil, and attaching the foil to the tungsten micromanipulator needle and then to an Omniprobe Cu lift-out grid. After the final FIB preparation step, the foils had thicknesses of 90–100 nm.

### Analytical methods

#### Chemical analysis

Backscattered electron images were obtained, and energy dispersive X-ray spectrometry (EDS) analyses of inclusions were performed, on a Jeol JXA 8530-F electron probe micro-analyser (EPMA). The chemical compositions of samples were determined by wavelength dispersive X-ray spectrometry (WDS) analysis using a Cameca SX100 EPMA. The accelerating voltage was 15 kV and the beam current was 10 nA. Samples were measured with a defocused beam (spot size at sample surface: 10 μm), to minimize the loss of F during analysis. The following natural and synthetic reference materials were used: F Kα PrF_3_; Na Kα albite; Si-*K*α sanidine; Ca- and P-*K*α fluorapatite; Fe-*K*α almandine; Sc-*K*α ScVO_4_; Y-*L*α YPO_4_; Sr-*L*α SrSO_4_; La-*L*α LaPO_4_; Ce-*L*α CePO_4_; Dy-*L*α DyPO_4_; Pr-*L*β PrPO_4_; Nd-*L*β NdPO_4_; Sm-*L*β SmPO_4_; Eu-*L*β EuPO_4_; Gd-*L*β GdPO_4_; Th-*M*α CaTh(PO_4_)_2_; Pb-*M*α vanadinite; U-*M*β metallic U. Prior to analysis, extended wavelength scans were done to recognise possible peak overlap. Raw X-ray intensities were corrected for matrix effects with a ϕρ(z) algorithm of X-PHI routine (Merlet 1994). An empirically determined correction factor was applied to the coincidence of 2nd-order of the Ce-*M*z with the F-*K*a line, and Dy-*L*α with Eu-*L*β line. Detection limits were calculated using Cameca’s Peaksight software, which is based on the method of Ziebold TO (1967). Further EPMA details are described elsewhere (Breiter et al. 2010; Škoda et al. 2015). The mineral formula calculation is based on the fixed number of Ca =1 atom per formula unit (apfu) lowered by the amount of Ca (quoted as Ca*) substituting for REE^3+^ to charge-compensate the entrance of Th^4+^ via substitution Ca_1_Th_1_REE_−2_. The amount of CO_2_ and OH was calculated based on the stoichiometry and electroneutrality. The assignment to mineral species was based on the (REE + Th + Ca*)/Ca ratio, where >1.875 corresponds to parisite–(Ce), 1.875 to 1.625 corresponds to unnamed *B*_3_*S*_4_ phase, and > 1.625 to 1.25 corresponds to röntgenite–(Ce).

Concentrations of rare-earth elements (REEs) were also determined by means of laser ablation–inductively coupled plasma–mass spectrometry (LA–ICP–MS) using a quadrupole Agilent 7500XE mass spectrometer equipped to an ESI NWR 193 excimer laser ablation system (193 nm wavelength). The LA–ICP–MS analyses were placed in close proximity to EPMA analysis points. The spot size was 75 μm with a repetition rate of 8 Hz (fluence of ~7 J/cm2). The helium carrier gas flow rate was ~0.75 l/min, 30 ms gas blank followed by 60 s of ablation and a dwell time of 30 ms for each individual mass. External independent calibration was done using NIST glass SRM610 and Ca as internal calibration element (Jochum et al. 2011). The USGS reference glass, BCR-2G and SRM612 glass were analysed as monitor standards (Rocholl 1998; Jochum et al. 2011). Data reduction was done using GLITTER 4.0 (Griffin et al. 2008).

#### Spectroscopy

Raman spectra and photoluminescence (PL) spectra were obtained at room temperature using two dispersive Horiba LabRAM HR800 and LabRAM HR Evolution spectrometers. Both systems have a focal length of 800 mm and are equipped with an Olympus BX series optical microscope, a diffraction grating with 1800 grooves per millimetre, and an Si-based, Peltier-cooled CCD detector. Spectra were excited with the 785 nm emission of a diode laser (PL), the 632.8 nm emission of a He-Ne laser (Raman and PL), the 532 nm emission of a frequency-doubled Nd:YAG laser (PL), and the 473 nm emission of a diode-pumped solid-state laser (PL). Laser energies on the sample surface were in the range 3–20 mW, which was well below the threshold of any absorption-induced sample changes. The spectral resolution for both systems was in the range 1.2 cm^−1^ (blue) to 0.7 cm^−1^ (near infrared). Spectra were obtained in the confocal mode, using a 100× objective (numerical aperture 0.9). The resulting lateral resolution was better than 1 μm and the depth resolution (with the laser focused at the sample surface) was ~2 μm. The system was calibrated using the Rayleigh line and Kr lamp emissions, resulting in a wavenumber accuracy better than 0.5 cm^−1^. All Raman and PL spectra were obtained in areas close to EPMA analysis points. It was ensured, however, that the distance between spectroscopic and chemical-analysis points was sufficiently large to avoid any artefact caused by the impact of the electron beam during EPMA analysis. Fitting of Raman spectra was done after appropriate background correction, assuming combined Lorentzian–Gaussian band shapes.

Optical absorption spectra were obtained using a Bruker IFS 66v/S Fourier-transform infrared spectrometer equipped with a mirror-optics IR-scope II microscope and a quartz beam splitter. A calcite Glan prism was used to polarise the light. Spectra were obtained at room temperature with two polarisations (E ⊥ c and E || c) in the range 25,000–5000 cm^−1^. The following combinations of light sources and detectors were used: Xe-lamp source and GaP detector for the spectral range 24,100–20,000 cm^−1^ (20 cm^−1^ spectral resolution; 1024 scans), W-lamp source and Si detector for the range 20,000–10,000 cm^−1^ (10 cm^−1^ spectral resolution; 1024 scans) and W-lamp source and Ge detector for the range 10,000–5200 cm^−1^ (10 cm^−1^ spectral resolution; 512 scans).

#### X-ray diffraction

Single-crystal X-ray diffraction was performed on a Stoe StadiVari system with open Eulerian cradle using a DECTRIS Pilatus 300 K detector with 450 μm Si layer and air-cooled Incoatec IμS 2.0 Mo micro-focus tube source. Measured fragments were approximately 60 × 60 × 60 μm^3^ [parisite–(Ce)] and 80 × 80 × 80 μm^3^ [röntgenite–(Ce)] in size, respectively. Frames were collected with angular steps of 0.5° [parisite–(Ce)] and 0.25° [röntgenite–(Ce)] in ω rotational mode. The sample-detector distance was set to 120 mm. The exposure time was set to 80 s [parisite–(Ce)] and 100 s [röntgenite–(Ce)] per frame. The measurements as well as integration, scaling and numerical absorption correction, were done with the X-AREA software collection 1.72 (STOE and Cie GmbH). More data collection parameters can be found in Table S1 in the Electronic Supplementary Material. All refinements were carried out using scattering curves from Prince (2004) and anisotropic displacement parameters for heavy atoms using SHELXL (Sheldrick 2015); the graphical user interface ShelXle (Hübschle et al. 2011) was used. The reciprocal lattice of the main and twin domains from the refined crystal structure (Table S3 in the Electronic Supplementary Material) of the present study was simulated with SingleCrystal 3.1.5 (CrystalMaker Software Ltd.).

#### Transmission electron microscopy

TEM investigations were performed using a TECNAI F20 XTWIN TEM operated at 200 kV with a field emission gun (FEG) as the electron source at the GFZ in Potsdam, Germany. The TEM is equipped with a Gatan Tridiem™ energy filter, an EDAX Genesis™ X-ray analyser with an ultra-thin window, and a Fishione high-angle annular dark field detector. A Tridiem energy filter was used for acquisition of bright and dark field images as well as high-resolution images applying a 20-eV window to the zero-loss peak. EDX spectra were acquired using the TIA software package in the scanning transmission mode of the TEM. To minimize mass loss due to electron sputtering during data acquisition the electron beam was scanned within a preselected area. The acquisition time of EDX spectra was 60 s.

## Results

### General mineralogical information

The samples’ mass densities range between 4.24 and 4.49 g/cm^3^, which corresponds reasonably well to published values of 4.30–4.39 g/cm^3^ (Flink 1901; Penfield and Warren 1899) and the theoretical “X-ray density” of 4.39 g/cm^3^. However, our results differ appreciably from the mass density of 3.79 g/cm^3^ reported by Guastoni et al. (2010).

Optical microscopy revealed the samples’ pseudo-uniaxial positive optical character. In crystals #1, #2 and #4, a number of mineral inclusions were found and identified by EDS and Raman measurements as dolomite, calcite, pyrite and quartz (Fig. 2). In crystal #1, calcite is intercalated within thin fluorcarbonate lamellae, which are oriented perpendicular to the *c* axis. All inclusions show considerably lower BSE intensities compared to their host parisite–(Ce), which is assigned to the considerably lower average atomic number of the inclusions.

All BSE images reveal strong striation of parisite–(Ce) parallel to the (1 1 0) plane, with periodic and aperiodic variations in BSE intensities and layer widths. The EPMA results (Table 1) indicate that the BSE intensity correlates with the REE/Ca ratio and the Th content. Hence, bastnäsite–(Ce) lamellae (determined by EDX analysis) are highest and röntgenite–(Ce) lamellae are lowest in BSE intensity.

**Table 1:** Results of EPMA chemical analyses and calculated mineral formulae for REE fluorcarbonates from the La Pita mine

Sample	Crystal #1			Crystal #2			Crystal #3			Crystal #4		
Number of analyses	9	10	15	8	10	6	7	4	7	5	7	6
Mineral^a^	Parisite–(Ce)	Parisite–(Ce)	Parisite–(Ce)	Parisite–(Ce)	Parisite–(Ce)	Parisite–(Ce)	Röngenite–(Ce)	? (presumable *B*_*3*_*S*_*4*_ phase)	Parisite–(Ce)	Röngenite–(Ce)	Parisite–(Ce)	Parisite–(Ce)
BSE intensity^b^	Low	Inter-mediate	High	Low	Inter-mediate	High	Low	Inter-mediate	High	Low	Inter-mediate	High
EPMA results (wt%)^c^:												
CO_2_^d^	24.3 ± 0.4	24.0 ± 0.8	24.0 ± 0.8	24.1 ± 0.8	24.4 ± 0.4	24.2 ± 0.4	25.4 ± 0.6	24.6 ± 0.1	24.6 ± 0.3	25.1 ± 0.4	24.2 ± 0.5	24.0 ± 0.2
F	6.86 ± 0.05	6.85 ± 0.11	6.86 ± 0.11	6.65 ± 0.10	6.6 ± 0.09	6.62 ± 0.10	6.54 ± 0.13	6.73 ± 0.16	6.91 ± 0.13	6.39 ± 0.16	6.85 ± 0.13	6.84 ± 0.11
CaO	10.4 ± 0.4	10.1 ± 0.4	10.1 ± 0.5	10.2 ± 0.4	10.5 ± 0.2	10.4 ± 0.2	12.65 ± 0.3	11.4 ± 0.1	10.7 ± 0.3	12.7 ± 0.4	10.4 ± 0.3	10.3 ± 0.1
Y_2_O_3_	0.88 ± 0.26	0.71 ± 0.12	0.64 ± 0.08	0.76 ± 0.12	0.83 ± 0.15	0.84 ± 0.20	1.03 ± 0.08	0.86 ± 0.09	0.77 ± 0.11	0.78 ± 0.18	0.64 ± 0.18	0.77 ± 0.10
La_2_O_3_	15.5 ± 0.5	14.3 ± 0.7	14.5 ± 0.8	14.4 ± 0.7	14.6 ± 0.6	14.4 ± 0.4	13.54 ± 0.3	14.0 ± 0.3	14.6 ± 0.6	13.0 ± 0.2	14.2 ± 0.3	13.8 ± 0.7
Ce_2_O_3_	27.7 ± 1.1	27.6 ± 1.1	27.3 ± 0.9	27.6 ± 1.0	27.6 ± 0.6	27.1 ± 0.9	26.1 ± 0.6	26.5 ± 0.6	27.4 ± 0.5	26.0 ± 0.2	27.6 ± 0.7	26.4 ± 0.6
Pr_2_O_3_	3.14 ± 0.26	3.37 ± 0.28	3.27 ± 0.50	3.22 ± 0.36	3.27 ± 0.37	3.20 ± 0.19	3.07 ± 0.22	3.24 ± 0.32	3.18 ± 0.26	3.09 ± 0.14	3.24 ± 0.36	3.31 ± 0.39
Nd_2_O_3_	10.4 ± 0.9	11.3 ± 0.5	11.5 ± 0.6	11.1 ± 0.4	11.1 ± 0.7	11.0 ± 0.6	10.7 ± 0.6	10.7 ± 0.2	11.0 ± 0.7	11.0 ± 0.6	11.3 ± 0.6	11.4 ± 0.6
Sm_2_O_3_	1.3 ± 0.21	1.42 ± 0.23	1.39 ± 0.27	1.46 ± 0.20	1.53 ± 0.27	1.46 ± 0.24	1.54 ± 0.33	1.37 ± 0.10	1.43 ± 0.20	1.39 ± 0.21	1.48 ± 0.34	1.44 ± 0.33
Eu_2_O_3_	0.22 ± 0.14	0.19 ± 0.22	0.14 ± 0.16	0.16 ± 0.10	0.13 ± 0.22	0.15 ± 0.15	0.11 ± 0.19	0.09 ± 0.15	0.13 ± 0.23	0.09 ± 0.16	0.14 ± 0.15	0.17 ± 0.19
Gd_2_O_3_	0.7 ± 0.30	0.72 ± 0.16	0.70 ± 0.32	0.78 ± 0.16	0.77 ± 0.12	0.83 ± 0.16	0.81 ± 0.21	0.83 ± 0.27	0.84 ± 0.15	0.79 ± 0.29	0.65 ± 0.20	0.83 ± 0.16
Dy_2_O_3_	0.22 ± 0.09	0.21 ± 0.11	0.21 ± 0.09	0.20 ± 0.12	0.25 ± 0.09	0.24 ± 0.13	0.28 ± 0.09	0.25 ± 0.09	0.23 ± 0.06	0.28 ± 0.09	0.26 ± 0.10	0.29 ± 0.16
ThO_2_	0.13 ± 0.14	0.53 ± 0.17	0.70 ± 0.41	0.54 ± 0.18	0.72 ± 0.22	1.23 ± 0.39	0.79 ± 0.10	0.90 ± 0.11	1.43 ± 0.14	0.24 ± 0.08	0.77 ± 0.35	1.36 ± 0.29
H_2_O^d^	0.12 ± 0.21	0.14 ± 0.29	0.13 ± 0.19	0.31 ± 0.22	0.43 ± 0.20	0.39 ± 0.20	0.19 ± 0.16	0.09 ± 0.21	0.18 ± 0.16	0.16 ± 0.16	0.14 ± 0.18	0.11 ± 0.11
F error	0.17	0.17	0.17	0.17	0.17	0.17	0.17	0.17	0.17	0.17	0.17	0.17
O=F	−2.89	−2.88	−2.89	−2.80	−2.78	−2.79	−2.75	−2.84	−2.91	−2.69	−2.88	−2.88
Total	99.0 ± 5.0	98.6 ± 5.2	98.6 ± 5.7	98.7 ± 4.9	100.0 ± 4.2	99.3 ± 4.3	100.0 ± 3.9	98.9 ± 2.9	100.5 ± 4.0	98.3 ± 3.3	99.0 ± 4.4	98.1 ± 4.0
Calculated mineral formulae (apfu)^e^:												
Ca	1.00	1.01	1.01	1.01	1.02	1.03	1.01	1.02	1.03	1.00	1.02	1.03
Y	0.04	0.04	0.03	0.04	0.04	0.04	0.04	0.04	0.04	0.03	0.03	0.04
La	0.51	0.50	0.50	0.49	0.49	0.49	0.37	0.43	0.49	0.35	0.48	0.48
Ce	0.91	0.94	0.94	0.93	0.91	0.92	0.71	0.81	0.90	0.70	0.92	0.90
Pr	0.10	0.11	0.11	0.11	0.11	0.11	0.08	0.10	0.10	0.08	0.11	0.11
Nd	0.34	0.38	0.39	0.37	0.36	0.36	0.28	0.32	0.35	0.29	0.37	0.38
Sm	0.04	0.05	0.05	0.05	0.05	0.05	0.04	0.04	0.04	0.04	0.05	0.05
Eu	0.01	0.01	0.00	0.01	0.00	0.00	0.00	0.00	0.00	0.00	0.00	0.01
Gd	0.02	0.02	0.02	0.02	0.02	0.03	0.02	0.02	0.03	0.02	0.02	0.03
Dy	0.01	0.01	0.01	0.01	0.01	0.01	0.01	0.01	0.01	0.01	0.01	0.01
Th	0.00	0.01	0.01	0.01	0.01	0.03	0.01	0.02	0.03	0.00	0.02	0.03
∑ REE	1.98	2.07	2.06	2.04	2.00	2.04	1.56	1.79	1.99	1.52	2.01	2.04
∑ cations	2.98	3.08	3.07	3.05	3.02	3.07	2.57	2.81	3.02	2.52	3.03	3.07
CO_3_^2−^	2.98	3.07	3.08	3.04	3.02	3.05	2.59	2.80	3.03	2.53	3.02	3.05
F	1.95	2.03	2.04	1.94	1.89	1.93	1.55	1.77	1.97	1.49	1.98	2.02
OH	0.04	0.04	0.04	0.10	0.13	0.12	0.05	0.02	0.05	0.04	0.04	0.03
∑ anions	4.97	5.14	5.16	5.08	5.04	5.10	4.19	4.59	5.05	4.06	5.04	5.10

Note: Na_2_O, SiO_2,_ P_2_O_5_, Sc_2_O_3,_ FeO, SrO, PbO and UO_2_ were also measured but averages were below the respective EPMA detection limit

^a^The assignment to mineral species is based on the (REE + Th + Ca*)/Ca ratio, where >1.875 corresponds to parisite–(Ce), 1.875 to 1.625 corresponds to unnamed *B*_*3*_*S*_*4*_ phase, and > 1.625 to 1.25 corresponds to röntgenite–(Ce)

^b^BSE intensities (based on individual contrast and brightness settings in the EPMA) refer to the respective crystal only and hence cannot be compared among samples

^c^Errors are quoted at the 2σ level

^d^CO_2_ and H_2_O were calculated from stoichiometry

^e^Calculated based on the assumption that Ca that is not charge-compensated by Th and U [according to (U,Th)^4+^ + Ca^2+^ ↔ 2REE^3+^] is 1.00 atoms per formula unit. Note that values quoted for röntgenite–(Ce) – ideal formula Ca_2_Ce_3_[(CO_3_)_5_F_3_] – correspond to half of the formula unit of this mineral and B_3_S_4_ phase Ca_4_Ce_7_[(CO_3_)_11_F_7_] – correspond to one quarter of the formula

**Fig. 3:**
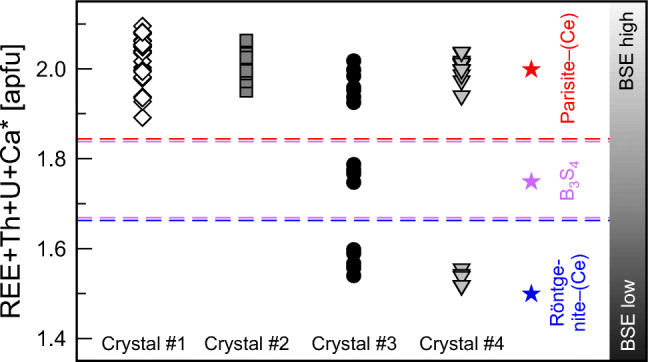
Plot of REE + Th + U + Ca* content of crystal #1–crystal #4 (EPMA data; Table 1), which correlate with the relative BSE intensity

All analysed fluorcarbonate phases are Ce dominant and therefore indicated by the suffix Ce added to the mineral name. Chemical analyses reveal three fluorcarbonate phases corresponding to distinct BSE intensities (Fig. 3). The brightest BSE area consists of parisite–(Ce) and the darkest BSE area consists of röntgenite–(Ce). Crystal #3 has been identified as an intermediate phase, whose BSE intensity and the ratio of (REE + Th + Ca*)/Ca (where Ca* is the amount of Ca that is needed for charge compensation of U + Th) in the formulae is between parisite–(Ce) and röntgenite–(Ce). The (REE + Th + Ca*)/Ca ratio of the intermediate phase (1.78–1.80) corresponds well to the ideal one (1.75) of the unnamed polysome *B*_*3*_*S*_*4*_, Ca_4_Ce_7_(CO_3_)_11_F_7_ (van Landuyt and Amelinckx 1975).

**Fig. 4:**
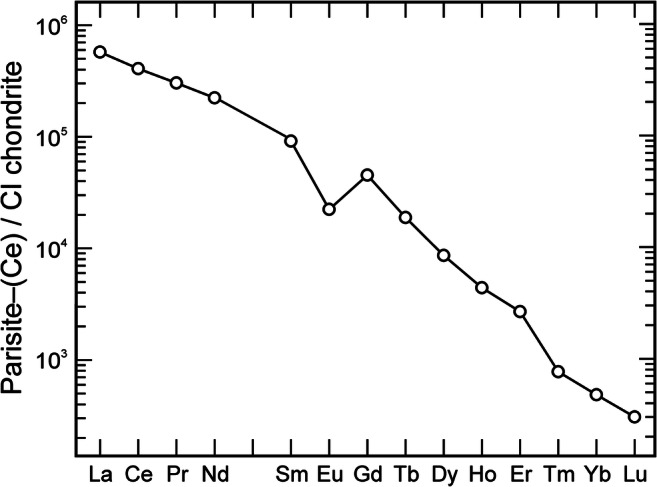
Mean CI chondrite-normalised REE concentrations in parisite–(Ce) crystals #1–#4 (LA-ICP-MS results; Table S2 in the electronic supplementary material). Sizes of symbols exceed the analytical uncertainties. Data show a decreasing trend from light to heavy REEs

The chondrite-normalised plot (Fig. 4) of REE concentrations as obtained by LA–ICP–MS (Table S2) indicates that there is virtually no Ce anomaly [Ce/Ce* = 0.97 for parisite–Ce and röntgenite–(Ce)] whereas a pronounced negative Eu anomaly exists [Eu/Eu* = 0.33 for parisite–Ce; Eu/Eu* = 0.31 for röntgenite–(Ce)]. All samples are highly enriched in light rare earth elements (LREE) and show a decreasing trend from lighter to heavier REEs (Fig. 4). This corresponds to results of Williams-Jones and Wood (1992) who found that fluorcarbonates are LREE-selective.

### Spectroscopic characterisation

#### Photoluminescence spectroscopy

**Fig. 5:**
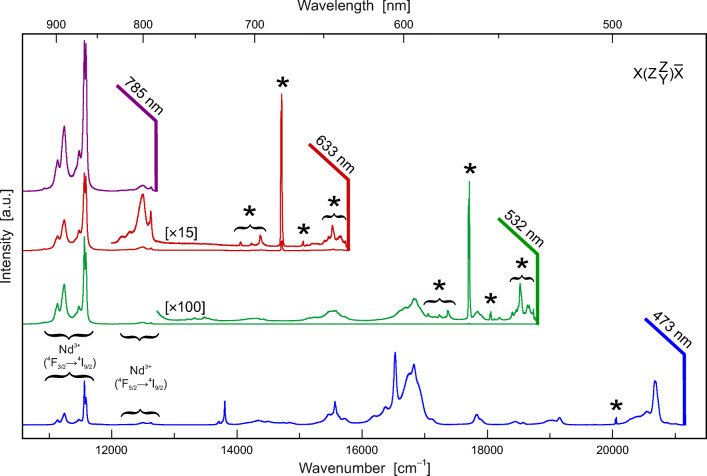
Spectra of parisite–(Ce) obtained with four different laser excitations. Note that the Raman patterns (marked with asterisks) are obscured, to vastly different degrees, by laser-induced PL. Whereas the 633 nm spectrum shows Raman bands in the 100–2000 cm^−1^ Raman shift range (which corresponds to 15,700–13,800 cm^−1^ absolute wavenumber) with minor luminescence background, the 473 nm and 785 nm spectra are heavily obscured by intense PL. Note that in the 633 nm Raman spectrum, the group of Nd^3+^-related emission lines at 12100–12650 cm^−1^ has apparent “Raman shifts” of 3150–3700 cm^−1^, which may easily be mistaken as “hydroxyl” Raman bands

Laser-induced PL spectra are shown in Fig. 5. They show groups of crystal-field-split emission bands (typical of REE elements with 4*f* electronic configuration) in the entire visible and the NIR (near infrared) range of the electromagnetic spectrum. The most prominent PL emission is due to the ^4^F_3/2_ → ^4^I_9/2_ electronic transition of Nd^3+^, which is observed in the range 11,600–11,000 cm^−1^ (corresponding to 860–910 nm wavelength; Fig. 5). The assignment of other REE-related emissions is still controversial and requires further investigation. We show PL spectra obtained with four laser excitations to underline the existing severe difficulties in obtaining a parisite–(Ce) Raman spectrum that is not biased by PL.

#### Raman spectroscopy

**Fig. 6:**
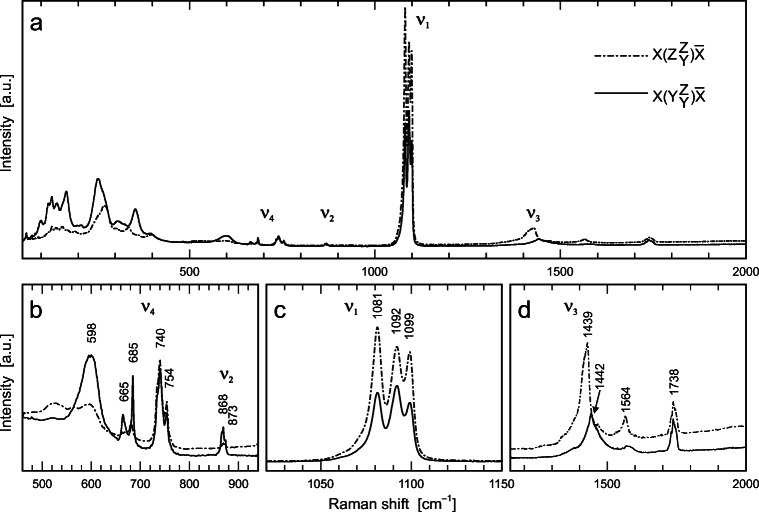
**a** Oriented Raman spectra of parisite–(Ce) obtained with 633 nm excitation. The scattering geometries are described using the so-called Porto notation (Damen et al. 1966). The assignment of internal CO_3_ vibrations (ν_1_–ν_4_) is based on White (1974). **b** Close-up showing the CO_3_ bending spectral range. The assignment of the broad signal at ~598 cm^−1^ is uncertain; it might either be a Raman band or, as an analytical artefact, caused by laser-induced luminescence. **c** Close-up showing the CO_3_ symmetric stretching range. The ν_1_ mode is split into three single bands. **d** Close-up showing the CO_3_ asymmetric stretching range

**Fig. 7:**
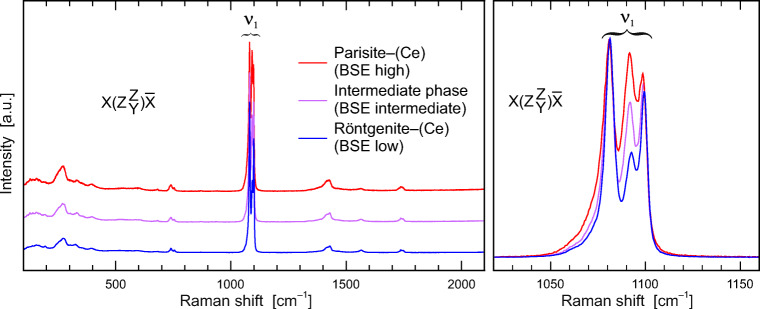
**a** Raman spectra of parisite–(Ce), röntgenite–(Ce) and the intermediate phase (tentatively assigned to an unnamed *B*_*3*_*S*_*4*_ phase), obtained with $\overrightarrow{E}$ // c, showing wide similarity of the principal spectral patterns. Spectra are shown with vertical arbitrary offset for clarity. **b** Close-up of the CO_3_ symmetric stretching range. Intensities are normalized to 100% of the highest signal. The tripartite ν_1_(CO_3_) band shows significant variations in relative intensities among the three phases

Raman spectra were obtained using 633 nm excitation. All other excitation wavelengths available in the present study (785 nm, 532 nm and 473 nm) have caused intense PL that, as an analytical artefact, strongly obscured the Raman spectrum (Fig. 5). Spectra are presented in Figs. 6 and 7. The orientation-dependence of Raman spectra obtained from crystal #2 is shown in Figs. 6a–d. Raman band intensities differ most significantly between spectra with the electric field vector ($\overrightarrow{E}$) polarized along the crystallographic *b* and *c* axis (shown in Figs. 6a–d). No obvious differences of Raman band intensities were detected in spectra from measurements with $\overrightarrow{E}$ aligned along *a* and *b* axis (not shown).

In Raman spectra of parisite–(Ce), we identified the four prominent internal vibrational modes of the carbonate anionic complex [ν_1_(CO_3_) – symmetric stretching vibration; ν_2_(CO_3_) – out-of-plane bending vibration; ν_3_(CO_3_) – antisymmetric stretching vibration; ν_4_(CO_3_) – in-plane bending vibration] according to analogue assignments of White (1974), Bischoff et al. (1985), and Gillet et al. (1996). Bands in the spectral region below 400 cm^−1^ are interpreted to be external lattice modes and are most likely biased by PL. Hence, band positions below 400 cm^−1^ were not labelled with Raman-shift values.

The ν_4_(CO_3_) Raman band in the range 665–754 cm^−1^, is apparently separated into two regions. The cause of the broad band at around 598 cm^−1^ is unclear; it may be caused by PL. The band assigned to the ν_2_(CO_3_) vibration is supposed to be in the region around 870 cm^−1^ and is clear visible with $\overrightarrow{E}$ polarised along the *b* axis. White (1974) assigned the ν_3_(CO_3_) vibration of the carbonate-ion in calcite to 1449 cm^−1^. In accordance, the parisite–(Ce) spectrum shows a strong band at ca. 1439 cm^−1^ ($\overrightarrow{E}$ aligned along the *c* axis) and 1442 cm^−1^ ($\overrightarrow{E}$ aligned along the *b* axis), which is notably orientation dependent. The assignment of two other strong asymmetric bands in this region at ca. 1564 cm^−1^ and 1738 cm^−1^ remains uncertain.

The most intense Raman band is assigned to the symmetric ν_1_(CO_3_) stretching vibration at ~1100 cm^−1^, which is split into three bands. Band positions are at ~1081 cm^−1^, ~1092 cm^−1^ and 1099 cm^−1^, which is consistent with observations of Wehrmeister et al. (2010). The intensity of the symmetric stretching vibration of the carbonate ion increases with $\overrightarrow{E}$ along the *c* axis. At a first glance, Raman spectra obtained from the three principal BSE areas (see Fig. 2) share principal similarities (Fig. 7a). A closer look, however, reveals that Raman spectra differ in intensity ratios of the tripartite carbonate bands around 1100 cm^−1^ (Fig. 7b). Note that Raman spectra were obtained orientation dependent. The full width at half maximum (FWHM) varies among the samples and within the crystal from high to low BSE intensities.

#### Optical absorption spectroscopy

**Fig. 8:**
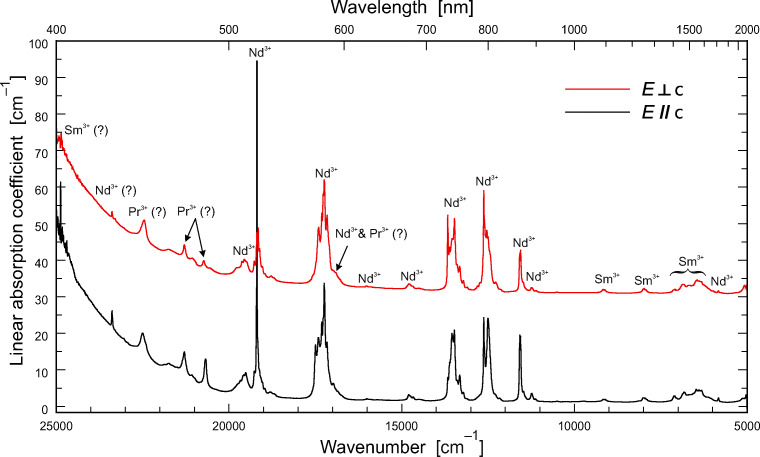
Polarised optical absorption spectra of parisite–(Ce) (sample thickness ~ 635 μm). Especially the sharp Nd^3+^–absorption band around 19,200 cm^−1^ shows noticeable orientation dependence

Optical absorption spectra were obtained parallel and perpendicular to the *c* axis (Fig. 8). Spectra consist of a large number of relatively sharp bands and an absorption edge in the UV spectral region, which slightly extends down to the blue region. Although parisite–(Ce) contains the entire range of lanthanides, only the LREEs Pr, Nd, and Sm could be assigned to distinct bands. Their absorbance does not show a strong dependence on the light polarisation with respect to the crystal orientation. Only the very sharp Nd absorption band around 19,200 cm^−1^ shows a noticeable orientation dependence.

### Structural characterisation

#### Single-crystal X-ray diffraction of parisite–(Ce)

The crystal structure of parisite–(Ce) was refined with Jana2006 (Petříček et al. 2014) to the space group *Cc* (no. 9) with lattice parameters of *a* = 12.30 Å, *b* = 7.10 Å, *c* = 28.25 Å and *β* = 98.5°, as proposed by Ni et al. (2000). The structural information is supplemented in Table S3.

**Fig. 9:**
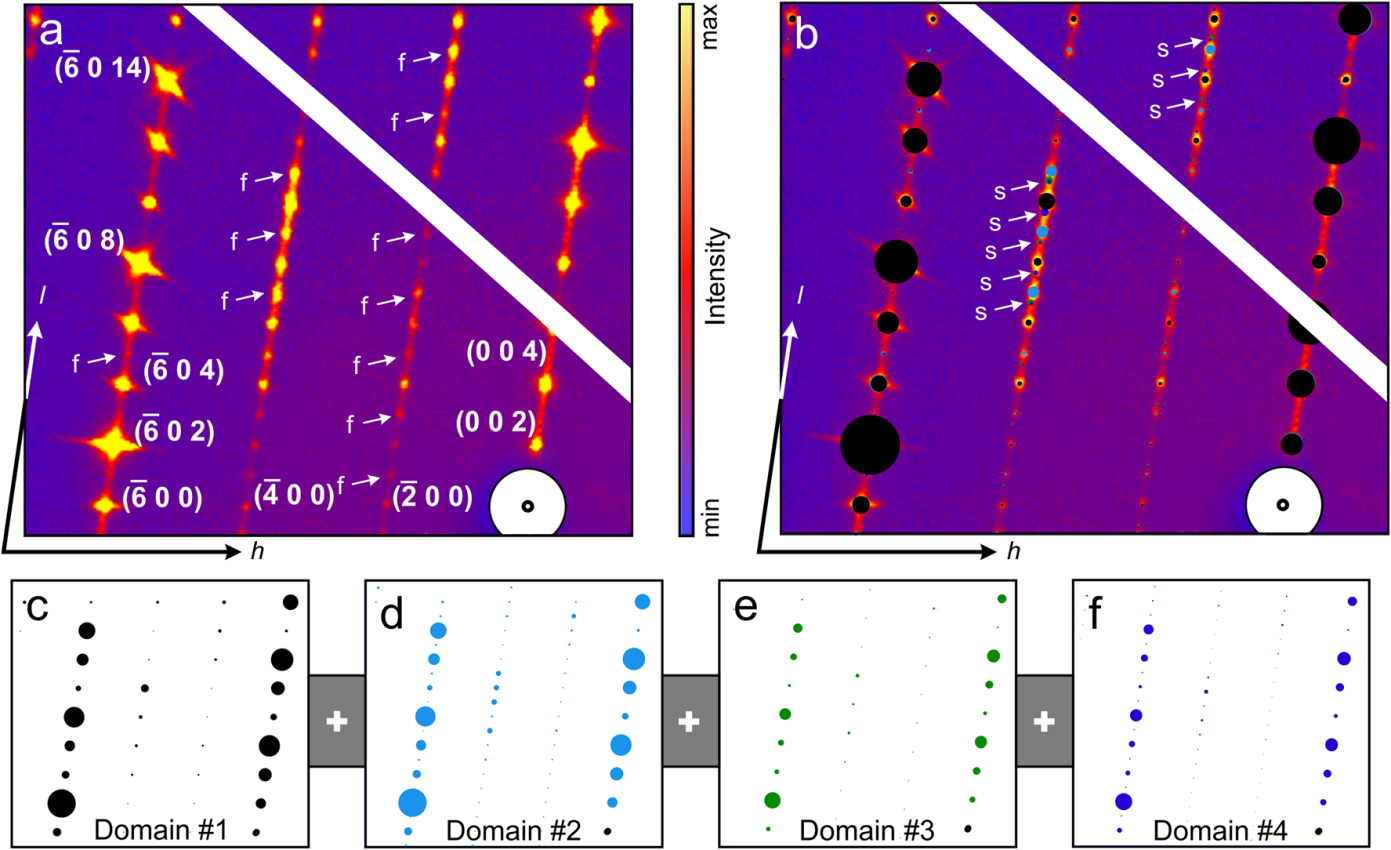
Reciprocal space map of parisite–(Ce) (calculated from results obtained from crystal #3). **a** Results. **b** Same, overlain by simulated reciprocal space patterns of four (twin) domains. The white diagonal line and the white circle represent a detector gap and the beam stop. Arrows labelled “f” mark forbidden peaks and arrows labelled “s” mark supercell peaks (if seen as part of domain #1). **c** (*h* 0 *l*) plane of the main domain. **d** (*h* $\overset{\hbox{--} }{h}$ *l*) plane of the twin, virtually breaking the *c*-glide plane. **e** (*h* 0 *l*) plane of the twin introducing the hexagonal supercell. **f** (*h* $\overset{\hbox{--} }{h}$ *l*) plane of combination twin

In contrast to Ni et al. (2000), the present study was taken up to 82° (2θ) with anisotropic displacement factors and occupancy refinement for heavy-atom positions. Subsequent twinning analysis was conducted, which was completely omitted by Ni et al. (2000) although indications of twinning have been reported. After a crystal structure check with checkCIF/PLATON (Spek 2009), 133 of 14,765 unique, not matching diffraction peaks with [F_obs −_ F_calc_ > 10Sig(F_obs_)] were omitted by a fitting routine under the assumption that these peaks were influenced by disregarded disorder effects, different from twinning (e.g. polysomatic disorder). It lowered the R_obs_ from ~7 to 4.79% and wR_all_ from ~15 to 12.45%. The crystal structure solution with a rhombohedral or trigonal symmetry and the hexagonal lattice parameters *a*, *b* = 7.11 Å and *c* ≈ 84.11 Å was not possible. Although several diffraction peaks simulating a supercell or commensurable modulation and multiple diffraction peaks forbidden in space group *Cc* are visible in the reciprocal space map along [0 0 *l*] (Figs. 9a–f).

We suggest that these diffraction peaks are due to multiple twinning. The latter is observable by searching for reticular twinning (using crystallographic programs such as ROTAX (Cooper et al. 2002) or Jana2006 (Petříček et al. 2014) during refinement. In Figs. 9a and b the stacking of the resulting reciprocal lattices of the main and three twin domains in the (*h* 0 *l*)-plane of the reciprocal space map is shown. The second and third twin domain (Figs. 9e and f, respectively) are considerably smaller than the main and the first twin domain (Figs. 9c and d, respectively).

**Fig. 10:**
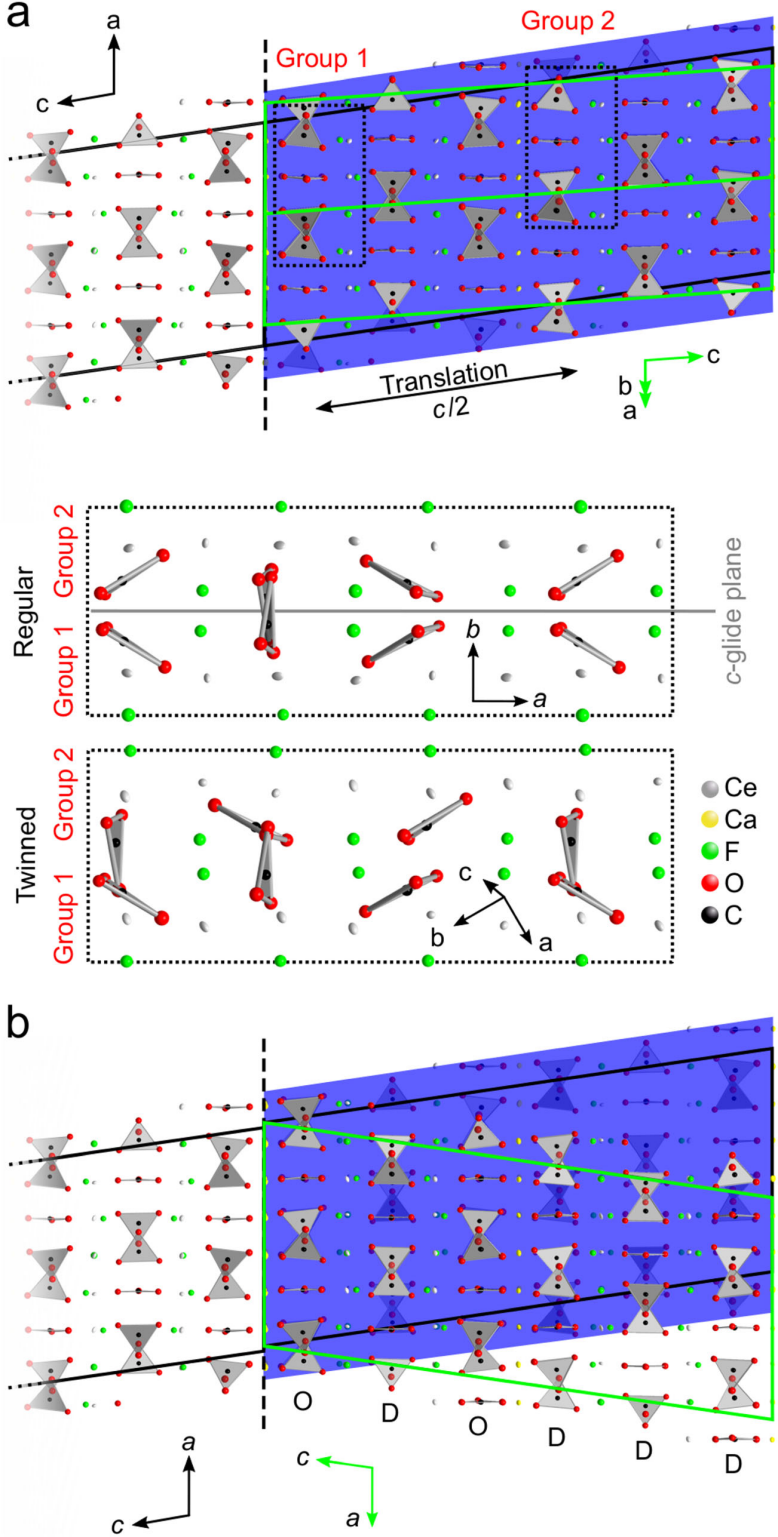
Sketches of twinned parisite–(Ce). Black dashed line marks twin boundary. **a** Twinning by reticular merohedry with a 180° rotation about $\left[\overline{1}\kern0.5em 1\kern0.5em 0\right]$. View near to [0 1 0] direction and [0 0 1], which shows best the virtual break of the c–glide plane (grey plane, mirroring over a–c plane). **b** Twinning by reticular pseudo-merohedry with apparent oblique (180° rotation about the a axis). View along the [0 1 0] direction, with marked CO_3_ group based order-disorder layers (O, D layers) match/mismatch between original (blue overlay) and twinned crystal structure

The first twin (domain #2 in Fig. 9d) is assigned to twinning caused by reticular merohedry (e.g. Herbst-Irmer 2016). The latter means that some reflections of domain #1 overlap diffraction peaks of domain #2, whereas other reflections may occur, where peaks should systematically absent. For example, the (2 $\overset{\hbox{--} }{2}$ 1) diffraction peak of the domain #2 and the (4 0 $\overset{\hbox{--} }{3}$) diffraction peak of the main domain #1 occur at the same position, whereby the (2 $\overset{\hbox{--} }{2}$ 1) peak is allowed and the (4 0 $\overset{\hbox{--} }{3}$) peak is forbidden due to the *c*-glide plane in space group *Cc*. Consequently, the *c*-glide plane of parisite–(Ce) is obscured in the diffraction pattern. The virtual break of the *c*-glide plane due to twinning by reticular merohedry is shown by mismatch of CO_3_-groups between the original and twinned crystal structure (Fig. 10a). Domain #2 is a 180° rotation in direct space around the rotation axis $\left[\overline{1}\kern0.5em 1\kern0.5em 0\right]$ (Fig. 10a) in relation to domain #1. Due to the rotation of the twin domain, a $\left(h\kern0.5em \overline{h}\kern0.5em \overline{l}\right)$-plane is visible instead of a (*h* 0 *l*)-plane (Fig. 9). The corresponding matrix is: *h*’ = 1/2 *h* − 1.5 *k*, *k*’ = − 1/2 *h* − 1/2 *k* and *l*’ = − 1/2 *h* + 1/2 *k* − *l*. Due to *C*-centering of the unit cell this type of twin pretend to be a merohedral twin. Twinning by merohedry means that all integer Miller indices are converted into other integer triplets, so that all reciprocal lattice points overlap (Parsons 2003). However, this symmetry is not reflected in the diffraction peak intensities.

The second twin (domain #3, Fig. 9e) is referred to as twinning caused by reticular pseudo-merohedry with apparent oblique or commonly called non-merohedral twin (e.g. Parsons 2003; Herbst-Irmer 2016; Petříček et al. 2016). Twinning matrices of this twin type contain irrational numbers. For prediction of overlaps, it is necessary to know not only the twinning matrices but also the actual setting of a measured reflection on the diffractometer. The twinning matrix for the non-merohedral twin domain #3 of parisite–(Ce) is mentioned below. Domain #3 can be explained as a two-fold twin in direct space around the rotation axis [1 0 0]. It rotates the twin domain in a way, that the (*h* 0 *l*)-plane is rotated about its normal by 180° with respect to domain #1. Due to the monoclinic angle (≈ 98.3°) some resulting diffraction peaks have fractional values for *l*, if they are considered as diffraction peaks of domain #1 (Fig. 9c). The resulting matrix is: *h*’ = *h*, *k*’ = − *k* and *l*’ = − 2/3 *h* – *l*, whereby (2*c*∙cos*β*)/*a* for parisite–(Ce) at room temperature and ambient pressure is close to 2/3. A non-merohedral twin law is commonly a symmetry operation causing a higher symmetry supercell (e.g. Parsons 2003; Petříček et al. 2016). Parisite–(Ce) can be transformed from the monoclinic unit cell to a hexagonal supercell with dimensions *a*, *b* = 7.11 Å and c ≈ 84.1 Å, as reported by Donnay and Donnay (1953). The corresponding transformation matrix is

$$ \left(\begin{array}{ccc}\raisebox{1ex}{$1$}\!\left/ \!\raisebox{-1ex}{$2$}\right.& \raisebox{1ex}{$1$}\!\left/ \!\raisebox{-1ex}{$2$}\right.& 0\\ {}\overline{\raisebox{1ex}{$1$}\!\left/ \!\raisebox{-1ex}{$2$}\right.}& \raisebox{1ex}{$1$}\!\left/ \!\raisebox{-1ex}{$2$}\right.& 0\\ {}1& 0& 3\end{array}\right). $$

Searching with Jana2006 for reticular twinning an even bigger hexagonal supercell with dimensions *a*, *b* = 14.2 Å and *c* ≈ 83.9 Å was found. Figure 10b visualises that the twinned and the non-twinned crystal structures show matching (ordered, O) and non-matching (disordered, D) CO_3_-group layers with the sequence (ODODDD DODODO DDDDOD, Fig. 10b). The pattern is repeated after three unit cells [3 × 28.25 × sin(98.32°) = 83.9 Å], producing the hexagonal supercell, which cannot be solved due to the not matching CO_3_-groups.

The third observed twin domain (domain #4, Fig. 9f) is assumed to be a combination of the first two twin laws. This combination is characterized by a rotation of 180° in direct space around the [$\overset{\hbox{--} }{1}$ 1 0] axis, followed by a 180° rotation about [1 0 0], which in sum can be interpreted as a six-fold rotation about [1 0 3]. Therefore, the reciprocal lattice of domain #4 is showing both, forbidden diffraction peaks and peaks with fractional values in *l*, when interpreted as diffraction peaks of domain #1 (Fig. 9c). The resulting twin law matrix is

$$ \left(\begin{array}{ccc}\raisebox{1ex}{$1$}\!\left/ \!\raisebox{-1ex}{$2$}\right.& 1.5& 0\\ {}\overline{\raisebox{1ex}{$1$}\!\left/ \!\raisebox{-1ex}{$2$}\right.}& \raisebox{1ex}{$1$}\!\left/ \!\raisebox{-1ex}{$2$}\right.& 0\\ {}\approx \raisebox{1ex}{$1$}\!\left/ \!\raisebox{-1ex}{$6$}\right.& \overline{\raisebox{1ex}{$1$}\!\left/ \!\raisebox{-1ex}{$2$}\right.}& 1\end{array}\right). $$

Streaking visible only along *h* 0 *l* rows, with *h* = ±│n∙3 + 1│and *h* = ±│n∙3 + 2│, is caused by the higher number of allowed diffraction peaks in the shown reciprocal planes of domain #4 and #2 and the misfit between “(2*c*∙cos*β*)/*a”* and “2/3” for domain #3 and #4. This selective streaking on *h* 0 *l* was already observed in HR–TEM study by Capitani (2019). Since the space group *Cc* is non-centrosymmetric, inversion twinning (a racemic twin), which is not visible in the diffraction pattern, due to only slight changes in the diffraction-peak intensities, was added. The racemic twin volume fraction refined to a positive value (≈10%) and slightly decreases the R_obs_. Hence it was kept in the refinement.

Searching for reticular twinning using the hexagonal supercell with dimensions *a*, *b* = 14.2 Å and *c* ≈ 83.9 Å in Jana2006 (Petříček et al. 2016), yielded twelve possible twin laws for the parisite–(Ce) measurement. All these twin laws belong to one of the twin laws mentioned above, just changing directions or combinations, but not all of them could be observed. Every twin domain that results in a negative or nearly zero volume fraction during refinement was neglected, which resulted in only four twin domains being fitted.

In the parisite–(Ce) single crystal X-ray diffraction analyses, no further modulation-vector and hence no incommensurate modulation could be found. However, instead of well observable diffraction peaks, continuous diffuse scattering is visible along all the reciprocal *h* 0 *l-*lattice rows, which means that apart from twinning, there is a certain one-dimensional disorder (stacking faults) present along the *c* axis. In contrast to the twin domains, this disorder affects all *h* 0 *l* rows (Fig. 9).

#### Single-crystal X-ray diffraction of röntgenite–(Ce)

The unravelling of the crystal structure of röntgenite–(Ce) is challenging, due to heavily twinning and the presence of complex stacking faults. The latter is visible from intense streaking and diffuse scattering along the *c*-direction in the reciprocal space map (Fig. S1). The unit-cell is found to be hexagonal with *a*, *b* = 7.14 and *c* = 69.82, as previously reported from Donnay and Donnay (1953) and Kasatkin et al. (2019). The space group is supposed to be *R*3, *R*$\overset{\hbox{--} }{3}$, *R*3*m* or *R*$\overset{\hbox{--} }{3}$*m*.

### Transmission electron microscopy

**Fig. 11:**
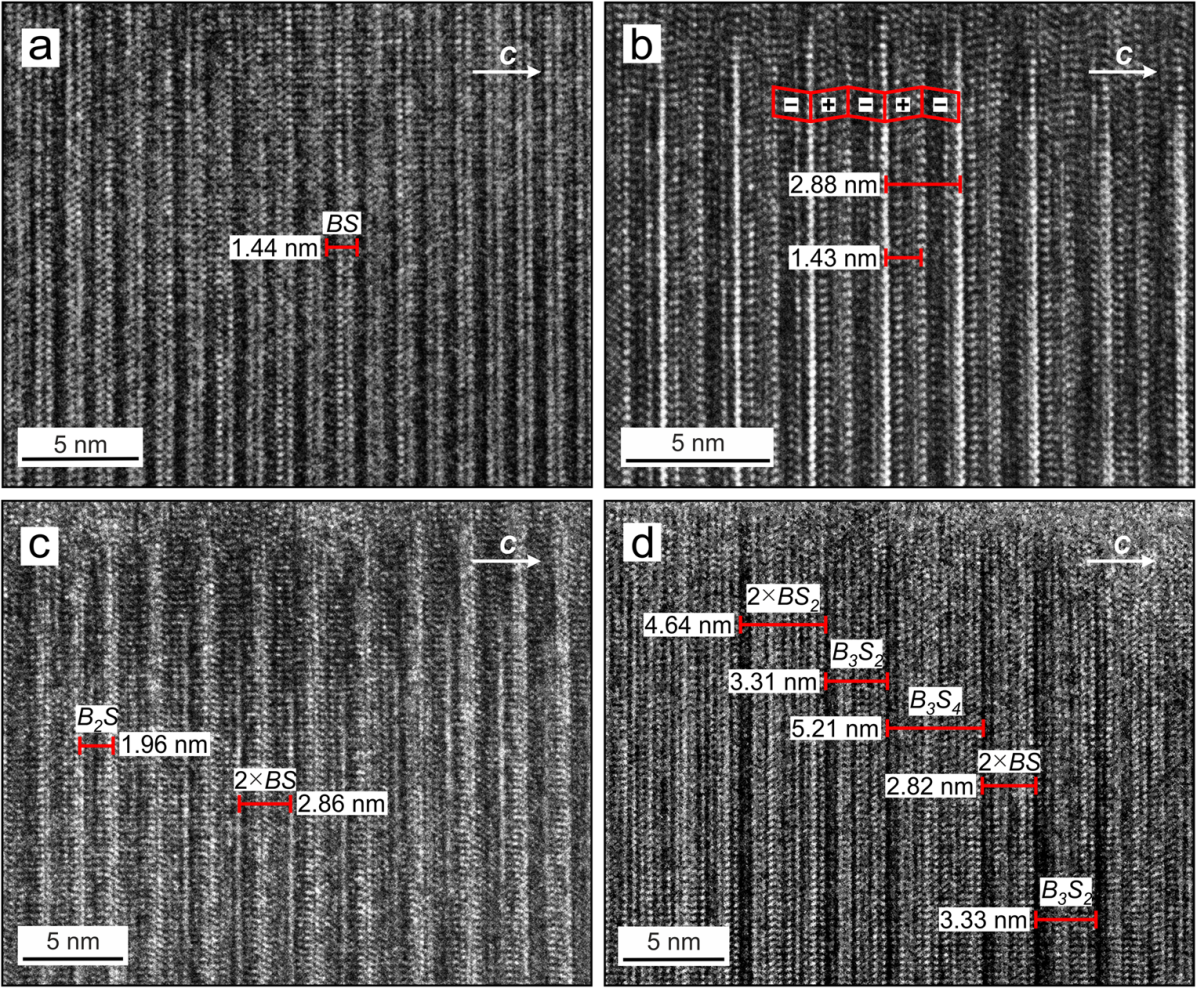
High resolution transmission electron microscopy (TEM) images of crystal #3 ([010] projection), visualizing ordered and disordered sequences in parisite–(Ce). Assignments of *B*–*S* slabs and mineral names are based on the observed lattice fringe spacings. **a** Typical well-ordered parisite–(Ce) sequences (*BS* or *VBB* or *dedgfg*) showing the common periodicity of ~14 Å. **b** Ordered parisite–(Ce) of recurrent packages with a thickness of ~28 Å. Red rhombic shapes show half-cells of parisite–(Ce) with alternating positive and negative slope. **c** Polysomatic disorder, which can be recognized from variations in lattice fringe spacing. Stacks with ~28 Å [parisite–(Ce)] and 19 Å [*B*_*2*_*S*: unnamed CaCe_3_(CO_3_)_4_F_3_] are observed. **d** Complex, polysomatically disordered structure, showing varying lattice fringe spacings of ~14 Å or ~ 28 Å [parisite(Ce)], ~33 Å [presumably *B*_*3*_*S*_*2*_: unnamed Ca_2_Ce_5_(CO_3_)_7_F_5_], ~46 Å [presumably röntgenite–(Ce)], and ~ 52 Å [presumably *B*_*3*_*S*_*4*_: unnamed Ca_4_Ce_7_(CO_3_)_11_F_7_]

High-resolution TEM images of parisite–(Ce) reveal remarkably complex stacking patterns, consisting of ordered and disordered sequences (Figs. 11a–d). Note that all of the stacking variations (polytypes or polysomes) discussed in the following were observed in the very same sample (Fig. 2, crystal #3). As mentioned above, the HR–TEM imaging technique is more sensitive to light elements such as C and O (Capitani 2019). TEM-images show that *d*-layers (REE-F) appear as darker lines, whereas *e*-layers (CO_3_) form a brighter line (thin; dotted) between two *d*-layers, and *f*-layers (Ca) are recognised as grey bands between bright lines (dotted) of *g*-layers (CO_3_) (cf. Capitani 2019).

Figures 11a, b show well-ordered stacking sequences of parisite–(Ce). The lattice fringe width is ~14 Å in Fig. 11a and ~ 28 Å in Fig. 11b. The former is interpreted to represent an ordered *BSBS* (or *dedgfgdedgfg*) layer sequence.

**Fig. 12:**
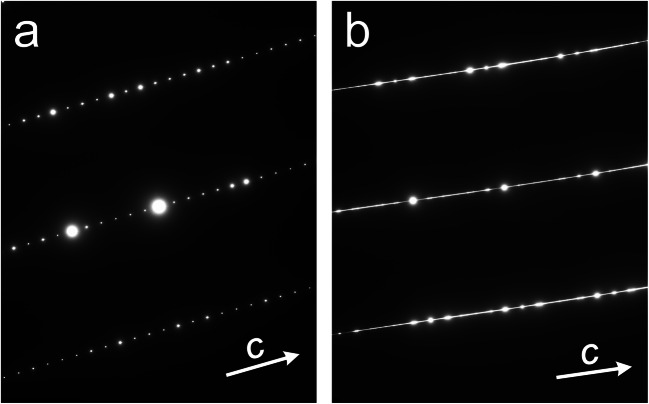
Selected area diffraction (SAED) patterns obtained from an ordered (**a**) and a disordered (**b**) domain. In the latter, streaking along the *c* direction demonstrates long-range stacking disorder

Figures 11c, d show polysomatically disordered parisite–(Ce). The stacking sequence shown in Fig. 11c consists of parisite–(Ce) (~28 Å) with an intercalated polysomatic fault that corresponds to the *B*_*2*_*S* polysome [CaCe_3_(CO_3_)_4_F_3_; ~19 Å]. The stacking sequence shown in Fig. 11d is most complex; it consists of a syntaxic intergrowth of several polysomes, recognisable from varying lattice fringe spacings. The fluorcarbonate phases present are assigned tentatively to parisite–(Ce), röntgenite–(Ce) [Ca_2_Ce_3_(CO_3_)_5_F_3_; ~46 Å], *B*_*3*_*S*_*4*_ [Ca_4_Ce_7_(CO_3_)_11_F_7_; ~52 Å] and *B*_*3*_*S*_*2*_ [Ca_2_Ce_5_(CO_3_)_7_F_5_; ~33 Å]. The existence of a *B*_*3*_*S*_*4*_ phase may be supported by EPMA results that indicate the presence of this fluorcarbonate phase in crystal #3 (there recognisable from its intermediate BSE intensity). However, it should be noted that EPMA results only reflect a linear combination of fine-scale disordered material whose average falls at *B*_*3*_*S*_*4*_. Sequential order or disorder is also visible in SAED patterns (Fig. 12). Whilst ordered domains yield sharp diffraction spots (Fig. 12a), SAED patterns of domains affected by long-range stacking disorder show pronounced streaking of diffraction spots (Fig. 12b) when viewed along <110> or [010] (cf. Capitani 2020).

## Discussion

### Evaluation of general mineralogical information

Mass density values of fluorcarbonate crystals in the present study differ appreciably from the mass density value reported by Guastoni et al. (2010). This may indicate that the sample studied by Guastoni et al. (2010) was not parisite–(Ce) but rather another fluorcarbonate mineral. The scatter of density values obtained in Guastoni et al. (2010) may be assigned to variations in chemical composition and/or the presence of inclusions and/or impurities.

Calculation of the mineral formulae reveals charge imbalance (Table 1), which might be assigned to F loss and/or rather to potential OH content during exposure to the electron beam. The latter interpretation is supported by results of Guastoni et al. (2009), who found significant amounts of hydroxylgroups in REE fluorcarbonates as well as existence of OH-dominated fluorcarbonates (e.g. hydroxylbastnäsite–(Ce). The absence of a Ce anomaly and the occurrence of a negative Eu anomaly may indicate formation of parisite–(Ce) under reducing conditions (cf. Hoskin and Schaltegger 2003), which in turn corresponds well with the reducing formation environment of black shales (Ottaway et al. 1994; Cheilletz and Giuliani 1996).

### Spectroscopic features

#### Analytical artefacts caused by PL emission

Raman spectra of fluorcarbonates need to be interpreted with caution, as the overlay with PL emissions (especially of REE) is likely (Fig. 5). This aspect is illustrated by the negative example of Raman interpretations by Frost and Dickfos (2007). These authors have investigated parisite–(Ce) and bastnäsite–(Ce) samples using 633 nm laser excitation and assigned bands that were recorded in the apparent Raman-shift range 3050–3800 cm^−1^ as OH-stretching Raman bands. In the present study, however, we have detected the ^4^F_5/2_ → ^4^I_9/2_ electronic transition of Nd^3+^ in the wavenumber range 12,750–12,000 cm^−1^ (or 785–833 nm wavelength), which only with 633 nm laser excitation corresponds to apparent Raman shifts of 3050–3800 cm^−1^. Also, the ^4^F_5/2_ → ^4^I_9/2_ Nd^3+^ emission shows crystal-field splitting into four Stark lines (Fig. 5). The spectral pattern of these four lines corresponds very well to the pattern (relative intensities and FWHMs) of the four “Raman bands” presented by Frost and Dickfos (Frost and Dickfos 2007; see Figs. 5a,b). This suggests that Frost and Dickfos (2007) have by mistake assigned emission lines as OH-related Raman bands.

Apart from potentially obscuring and biasing Raman spectra, PL emissions may prove useful in mineral identification. The PL spectra of parisite–(Ce) presented in the present study may assist in verifying the identity of this mineral, using the characteristic emission “fingerprint” that is due to the particular crystal-field splitting of Nd^3+^-related electronic transitions in parisite–(Ce) (cf. Lenz et al. 2013; Zeug et al. 2017).

#### Evaluation of Raman spectroscopic data

Guastoni et al. (2009) determined positions of the ν_1_(CO_3_) band of a parisite–(Ce) crystal at ~1083, 1093 and 1101 cm^−1^, which is remarkably different to ν_1_(CO_3_) band positions obtained from fluorcarbonate minerals of the present study. Obvious differences in spectral band positions from values reported by Guastoni et al. (2009), may be due to a strongly deviating composition of their parisite–(Ce) sample that possibly represents another fluorcarbonate species (see above discussion on differences in their obtained sample mass density).

Variations of the FWHMs of the tripartite ν_1_(CO_3_) band among the samples and within the crystal from high to low BSE intensities might be due to differences in the crystal chemical composition. Composition dependent changes of the FWHM and band positions have already been reported from magnesian calcites with varying Mg contents (Bischoff et al. 1985). Moreover, heating experiments do not result in any notable decrease of the FWHMs. Therefore, a band broadening due to radiation damage can be excluded.

#### Non-destructive identification of parisite–(Ce) and röntgenite–(Ce)

Different carbonate minerals can be distinguished based on their ν_1_(CO_3_) Raman band(s). The number(s) and spectral position(s) of these Raman bands depend, among other factors, on the cations neighbouring the carbonate groups (ionic radius, valence) and the coordination in the crystal structure. For instance, calcite and magnesium calcite show one single ν_1_(CO_3_) band that, however, differ in spectral positions. Increasing substitution of Mg in calcite results in increased Raman-shift values of the ν_1_(CO_3_) band, which is accompanied by simultaneous gain of its FWHM (Bischoff et al. 1985). Another example relates to fluorcarbonate-series minerals: Bastnäsite–(Ce) can easily be distinguished from parisite–(Ce), röntgenite–(Ce) and synchysite–(Ce), because the latter three fluorcarbonate minerals show a splitting of the ν_1_(CO_3_) band at around 1100 cm^−1^, whereas bastnäsite–(Ce) does not (Yang et al. 2008; Kasatkin et al. 2019).

**Fig. 13:**
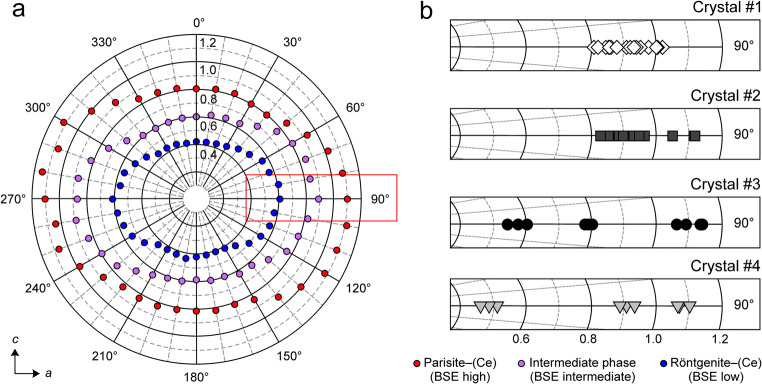
Ratio of the heights of the 1092 cm^−1^ and 1081 cm^−1^ Raman bands. **a** Circular plot of the 1092 cm^−1^/1081 cm^−1^ band-height ratio against the angle between the $\overrightarrow{E}$ polarization of the incident laser light and the sample’s crystallographic *c* axis, obtained from three interior regions in crystal #3 with 10° steps. **b** Analogous close-up plots (horizontally stretched) corresponding to the red rectangle in subfigure a, showing band-height ratios obtained with $\overrightarrow{E}$ ⊥ *c* in other regions of crystal #3 and from the other three samples. Note that with $\overrightarrow{E}$ ⊥ *c*, a 1092 cm^−1^/1081 cm^−1^ band-height ratio of <0.65 indicates röntgenite–(Ce)

As stated above, parisite–(Ce) crystal #3 shows three principal areas that differ in BSE intensity (see Fig. 2). Chemical analyses and single-crystal X-ray data reveal that low BSE intensities correspond to röntgenite–(Ce), areas with high BSE intensities correspond to parisite–(Ce) and an interjacent unnamed phase is characterized by intermediate BSE intensity. As changes of the polarisation direction of the laser beam relative to the crystal orientation results in changes of the intensity ratios of the three bands of the ν_1_(CO_3_) vibration (Fig. 7b), the latter may be used as an indicator to distinguish the fluorcarbonate phases corresponding to the three intensity BSE zones (bright, intermediate and dark zone see crystal #3 in Fig. 2). Figs. 13a and b demonstrate Raman-band-intensity ratios of 1092 cm^−1^/1081 cm^−1^, which slightly depend on the orientation of the crystal with respect to the laser beam polarization ($\overrightarrow{E}$). With $\overrightarrow{E}$ parallel to the *c* axis (Fig. 13a) röntgenite–(Ce) has a minimum band ratio of 0.41. With $\overrightarrow{E}$ aligned parallel to the *a* axis (Fig. 13a), röntgenite–(Ce) has a maximum band ratio of 0.61. Likewise, the ν_1_(CO_3_) band ratio, indicative for parisite–(Ce), varies between 1.05 (minimum) and 1.15 (maximum). This Raman spectroscopic discrimination may be advantageous, if a fast and/or non-destructive identification is required. As there is a considerable lack of information about röntgenite–(Ce) in the literature, we suppose that this rapid mineral identification tool could foster research on this mineral phase. However, TEM images reveal that the fine-scale intergrowth of different fluorcarbonate phases is visible down to the nanometre scale. In contrast, Raman spectroscopy is a method on the micrometre scale and the instrumental settings define the analysis volume which was about 3 μm^3^ in the present study. Hence, Raman spectra may provide information of bulk composition of nanometre-sized fine-scale intergrowth of different fluorcarbonate phases from time to time. The proposed fluorcarbonate-mineral identification by means of the intensity ratio of the tripartite carbonate band is only considered as a first approach. It is clear that the applicability of this method needs to be supported by reference analyses of an extended set of fluorcarbonate samples. In addition, the capability to distinguish other fluorcarbonate species with a tripartite ν_1_ (CO_3_) band from parisite–(Ce) and/or röntgenite–(Ce) by this methodology needs further verification.

### Interpretation of crystal structural data

#### Single-crystal X-ray diffraction of parisite–(Ce)

The crystal structure check results in a misfit with several non-matching diffraction peaks. Such a misfit may be caused by another stacking arrangement of the same compositional layer. For example, the stacking sequence *VBVBBB* (*BBSS*) of parisite–(Ce) with space group *C*1, has been found in a recent HR–TEM study by Capitani (2019). The latter may form lamellae within the most common polytype of parisite–(Ce) [space group *Cc* with stacking sequence *VBBVBB* (*BSBS*)] without causing notable strain. It is most likely that such a polytype is also present in the sample studied here.

#### Single-crystal X-ray diffraction of röntgenite–(Ce)

Due to intense streaking and diffuse scattering along the *c*-direction (Fig. S1 in the Electronic Supplementary Material) the structure of röntgenite–(Ce) could not be reliably solved. For instance, in the (*h* 0 *l*)-plane of the calculated reciprocal space map (Fig. S1) diffraction peaks are not visible at *h* = ±│n∙3 + 1│and h = ±│n∙3 + 2│, except for very broad peaks around (2 0 $\overset{\hbox{--} }{18}$), ($\overset{\hbox{--} }{2}$ 0 $\overset{\hbox{--} }{18}$) and (2 0 18).

In comparison with the reciprocal space map of parisite–(Ce) (Fig. 9), röntgenite–(Ce) shows four diffraction peaks between the main reflections of the *h* 0 *l*-rows with *h* = ±│n∙3│. The latter suggest a subcell with *a*, *b* = 7.14 Å and *c* = 14 Å. Hence, a monoclinic unit cell could be found for röntgenite–(Ce) with *a* = 12.30 Å, *b* = 7.12 Å, *c* = 23.54 Å and *β* = 100.1°. However, for this monoclinic cell the crystal structure solution was not successful. As mentioned above, diffraction pattern of reticular twins can be indexed in a supercell. As shown in Fig. S1 diffuse scattering and broad, weak diffraction peaks of the sample impede the unravelling of potential polysomatically and polytypical disorder.

## Transmission electron microscopy

Although the corresponding parisite–(Ce) polytype is most commonly documented in the literature, it is, however, inconsistently described. For instance, Meng et al. (2001a, 2001b) assigned it to the 6*R*_1_ polytype whereas Ni et al. (2000) and Capitani (2019) assigned it to the 2*M*_1_ polytype. The latter seems to be more appropriate, as the parisite–(Ce) lattice has monoclinic symmetry. The assignment of the second polytype of parisite–(Ce) (Fig. 11b) remains unclear. Unfortunately, due to its limited spatial resolution, the lattice fringe image cannot be reliably compared in detail with the TEM images of Capitani (2019; 2*M*_2_ polytype) and Meng et al. (2001b; 6*R*_2_ polytype). For the same reason, we cannot obtain reliable information on the arrangement of sub-halfcell fringes, and whether or not halfcells (~14 Å) have developed polytypic disorder.

## Conclusions

Parisite–(Ce) from the La Pita mine, Colombia, shows polytypic and polysomatic variability of layer sequences within the structure. Twinning, polytypic and polysomatic disorder of syntaxic intergrowth impede the crystal structure solution. However, our data imply a monoclinic crystal structure with lattice parameters *a* = 12.30 Å, *b* = 7.10 Å, *c* = 28.25 Å and *β* = 98.3° and the space group *Cc* for parisite–(Ce). The crystal structure refinement of röntgenite–(Ce) was not possible, due to intense twinning and the presence of complex stacking faults.

Two ordered, clearly distinguishable polytypes of parisite–(Ce) were observed in HR–TEM images. The predominant polytype is assigned to the most common 2*M*_1_ parisite–(Ce), 2*M*_1_, which is identical to the 6*R*_1_ polytype reported by Meng et al. (2001b). Reliable assignment of the second polytype was not possible. In polysomatically disordered sequences, five polysomes were observed that presumably correspond to parisite–(Ce), röntgenite–(Ce), *B*_*2*_*S*, *B*_*3*_*S*_*2*_, and *B*_*3*_*S*_*4*_. Chemical data obtained from the intermediate BSE phase support the assumption of the occurrence of a *B*_*3*_*S*_*4*_ phase. In combination with EPMA data, BSE images reveal that BSE intensities correlate with the Ca content in REE-fluorcarbonate minerals. Both BSE and TEM images show that the Maripí parisite–(Ce) is decidedly heterogeneous, with fine-layered zoning perpendicular to the *c* axis.

Raman spectra of parisite–(Ce) and röntgenite–(Ce) are widely similar. However, they are distinguishable from the intensity ratios of the tripartite carbonate band [ν_1_(CO_3_), symmetric stretching vibration] around 1100 cm^−1^, although the ν_1_(CO_3_) band is orientation-dependent. It was possible to distinguish parisite–(Ce) from röntgenite–(Ce) using the 1092 cm^−1^/1081 cm^−1^ band intensity ratio provided that oriented spectra were obtained. Although we managed to discriminate fluorcarbonate phases based on Raman spectra, further investigations are needed to support our observations.

Optical absorption and laser-induced PL spectra of the Maripí parisite–(Ce) are dominated by various absorptions and emissions of REEs, respectively. This observation was not unexpected, as REEs are commonly enriched in fluorcabonate phases. Strong laser-induced REE emissions hamper the Raman analysis of parisite–(Ce); reliable spectra could only be obtained with 633 nm laser excitation.

Corresponding with earlier findings, the present study shows that each fluorcarbonate is unique regarding its stacking pattern and may consist of various intergrowths of several fluorcarbonate phases. It is hence difficult to assign fluorcarbonate samples to one particular fluorcarbonate mineral, whereas the use of a more general term such as “fluorcarbonate polycrystal” appears more appropriate in most cases. However, many authors prefer to name their samples according to the main component, which is parisite–(Ce) in our case.

## Electronic supplementary material

ESM 1 (DOCX 178 kb)

ESM 2 (TIF 14084 kb)

High Resolution image (PNG 662 kb)
