# Supplementary material for: The parisite–(Ce) enigma: challenges in the identification of fluorcarbonate minerals
Source: Mineral Petrol. 2020 Oct 10;115(1):1–19. doi: 10.1007/s00710-020-00723-x (PMC7831744; doi:10.1007/s00710-020-00723-x)
Supplement: Supplementary file 1 — (DOCX 178 kb) [file 710_2020_723_MOESM1_ESM.docx]

**Electronic Supplementary Material**

accompanying the article

**“The parisite–(Ce) enigma: Problems in the identification of fluorcarbonate minerals, and a new method to distinguish non-destructively parisite–(Ce) and röntgenite–(Ce)”**

by Manuela Zeug, Lutz Nasdala, Martin Ende, Gerlinde Habler, Christoph Hauzenberger, Chutimun Chanmuang N., Radek Škoda, Dan Topa, Manfred Wildner and Richard Wirth

**Table S1** Data collection parameters and unit cell and refinement results for parisite–(Ce) and röntgenite–(Ce)

| Phase | Parisite–(Ce) | Röntgenite–(Ce) |
| --- | --- | --- |
| Space group (number) | *Cc* (9) | *R*3*?* |
| *a* (Å) | 12.30(1) | 12.30(1) |
| *b* (Å) | 7.10(1) | 7.12(1) |
| *c* (Å) | 28.25(1) | 23.54(1) |
| *β* (°) | 98.32(1) | 100.1(1) |
| V (Å³) | 2445.0(1) | 2029.6(1) |
| Z | 4 | 8 |
|  |  |  |
| Measured reflections | 37166 | 10899 |
| Unique reflections (observed ; all) | 12723 ; 14765 | 570 ; 675 |
| Maximum 2θ (°) | 81.98 | 69.51 |
| R_int_ ; R_sigma_ | 0.022 ; 0.012 | 0.029 ; 0.013 |
|  |  |  |
| R1(observed)^a^ ; R1(all) | 0.047 ; 0.056 | – |
| wR2 | 0.122 | – |
| Goodness of fit | 2.67 | – |
| Number of refined parameters | 261 | – |
| Maximum ; minimum e^–^ density (e^–^ Å^-3^) | 3.56 ; -3.69 | – |

^a^ observed: I>3σ(I)

**Table S2** Results of LA–ICP–MS chemical analyses for REE fluorcarbonates from the La Pita mine

| Sample number | Crystal #1 |  |  | | | Crystal #2 | |  | |  | | Crystal #3 | |  | |  | | Crystal #4 |  | |  | |
| --- | --- | --- | --- | --- | --- | --- | --- | --- | --- | --- | --- | --- | --- | --- | --- | --- | --- | --- | --- | --- | --- | --- |
| Number of spots | 2 | 2 | 4 | | | 3 | | 2 | | 3 | | 3 | | 2 | | 3 | | 2 | 3 | | 3 | |
| Mineral | Parisite–(Ce) | Parisite–(Ce) | Parisite–(Ce) | | | Parisite–(Ce) | | Parisite–(Ce) | | Parisite–(Ce) | | Röngenite–(Ce) | | Parisite–(Ce) | | Parisite–(Ce) | | Röngenite–(Ce) | Parisite–(Ce) | | Parisite–(Ce) | |
| BSE intensity | Low | Intermediate | High | | | Low | | Intermediate | | High | | Low | | Intermediate | | High | | Low | Intermediate | | High | |
| Element^a^ | LA–ICP–MS results (ppm)^b^ | | |  |  | |  | |  | |  | |  | |  | |  | | |  | |  |
| Li (7) | 17.7 ± 8.4 | 0.47 ± 0.86 | 0.24 ± 0.38 | | | 0.19 ± 0.19 | | 1.05 ± 0.06 | | 2.12 ± 0.21 | | 1.01 ± 1.08 | | 0.6 ± 0.1 | | 0.62 ± 0.50 | | 1.55 ± 1.30 | 1.63 ± 4.76 | | 0.84 ± 0.20 | |
| Be (9) | 0.01 ± 0.04 | 0.01 ± 0.02 | 0.01 ± 0.03 | | | 0.03 ± 0.01 | | 0.01 ± 0.04 | | 0.01 ± 0.03 | | 0.03 ± 0.01 | | n.d. | | 0.01 ± 0.03 | | 0.01 ± 0.04 | 0.01 ± 0.04 | | 0.02 ± 0.03 | |
| AL (l27) | n.d. | n.d. | 0.01 ± 0.05 | | | 0.02 ± 0.07 | | 0.06 ± 0.00 | | 0.04 ± 0.07 | | 0.04 ± 0.07 | | 0.06 ± 0.01 | | 0.04 ± 0.07 | | n.d. | 0.02 ± 0.08 | | n.d. | |
| Si (29) | 786 ± 1454 | 260 ± 73 | 260 ± 44 | | | 240 ± 46 | | 238 ± 53 | | 234 ± 29 | | 445 ± 638 | | 217 ± 23 | | 243 ± 3 | | 257 ± 26 | 249 ± 81 | | 246 ± 63 | |
| P (31) | 6.49 ± 4.60 | 8.39 ± 0.85 | 9.55 ± 0.66 | | | 9.16 ± 1.40 | | 8.16 ± 0.34 | | 8.13 ± 2.48 | | 7.71 ± 0.95 | | 8.50 ± 0.23 | | 8.96 ± 1.21 | | 10.9 ± 2.0 | 10.4 ± 1.92 | | 8.9 ± 1.17 | |
| Ca (43) | 78900 ± 2330 | 77200 ± 202 | 77000 ± 845 | | | 76900 ± 515 | | 78100 ± 505 | | 77300 ± 2350 | | 94400 ± 437 | | 84700 ± 910 | | 77300 ± 286 | | 94200 ± 1420 | 77700 ± 623 | | 77700 ± 218 | |
| Ti (49) | 0.05 ± 0.14 | n.d. | n.d. | | | n.d. | | n.d. | | n.d. | | n.d. | | n.d. | | n.d. | | 0.11 ± 0.31 | n.d. | | n.d. | |
| V (51) | n.d. | 0.99 ± 2.78 | n.d. | | | n.d. | | n.d. | | 0.01 ± 0.00 | | 0.01 ± 0.00 | | 0.01 ± 0.00 | | n.d. | | n.d. | 0.01 ± 0.01 | | n.d. | |
| Cr (53) | n.d. | n.d. | 0.04 ± 0.16 | | | n.d. | | 0.17 ± 0.05 | | 0.06 ± 0.22 | | n.d. | | n.d. | | 0.13 ± 0.24 | | n.d. | 0.11 ± 0.20 | | 0.07 ± 0.24 | |
| Mn (55) | 6.01 ± 0.41 | 4.86 ± 0.42 | 4.71 ± 0.44 | | | 5.47 ± 0.10 | | 6.66 ± 0.08 | | 5.95 ± 0.85 | | 7.22 ± 0.33 | | 5.96 ± 0.17 | | 5.14 ± 0.28 | | 6.65 ± 0.79 | 4.69 ± 0.51 | | 4.56 ± 0.13 | |
| Co (59) | 0.09 ± 0.02 | 0.07 ± 0.03 | 0.07 ± 0.01 | | | 0.06 ± 0.01 | | 0.06 ± 0.01 | | 0.06 ± 0.01 | | 0.08 ± 0.06 | | 0.06 ± 0.00 | | 0.06 ± 0.02 | | 0.06 ± 0.01 | 0.05 ± 0.01 | | 0.06 ± 0.00 | |
| Ni (60) | 0.81 ± 0.06 | 0.78 ± 0.07 | 0.72 ± 0.08 | | | 0.73 ± 0.05 | | 0.77 ± 0.07 | | 0.7 ± 0.08 | | 0.88 ± 0.13 | | 0.76 ± 0.04 | | 0.68 ± 0.10 | | 0.85 ± 0.17 | 0.67 ± 0.11 | | 0.68 ± 0.03 | |
| Cu (65) | 0.12 ± 0.02 | 0.07 ± 0.01 | 0.05 ± 0.11 | | | 0.1 ± 0.04 | | 0.06 ± 0.16 | | 0.06 ± 0.11 | | 0.05 ± 0.09 | | 0.04 ± 0.12 | | 0.03 ± 0.09 | | 0.05 ± 0.14 | 0.06 ± 0.10 | | 0.06 ± 0.10 | |
| Zn (66) | 0.03 ± 0.09 | 0.07 ± 0.01 | 0.05 ± 0.07 | | | 0.07 ± 0.00 | | 0.03 ± 0.08 | | 0.04 ± 0.08 | | n.d. | | n.d. | | n.d. | | 0.04 ± 0.10 | 0.02 ± 0.07 | | 0.02 ± 0.08 | |
| Ga (71) | 1783 ± 55 | 1813 ± 40 | 1760 ± 43 | | | 1761 ± 66 | | 1718 ± 56 | | 1684 ± 118 | | 1663 ± 20 | | 1644 ± 88 | | 1661 ± 23 | | 1638 ± 47 | 1654 ± 78 | | 1664 ± 35 | |
| Ge (74) | 153 ± 6.26 | 166 ± 0.01 | 167 ± 9.45 | | | 164 ± 6.40 | | 161 ± 7.27 | | 158 ± 12.2 | | 160 ± 4.44 | | 153 ± 8.71 | | 154 ± 3.47 | | 157 ± 1.58 | 153 ± 6.78 | | 165 ± 3.00 | |
| Rb (85) | 0.31 ± 0.06 | 0.22 ± 0.05 | 0.19 ± 0.04 | | | 0.23 ± 0.07 | | 0.26 ± 0.02 | | 0.24 ± 0.01 | | 0.29 ± 0.08 | | 0.26 ± 0.05 | | 0.25 ± 0.08 | | 0.32 ± 0.10 | 0.2 ± 0.04 | | 0.27 ± 0.07 | |
| Sr (88) | 37.2 ± 2.0 | 34.2 ± 13.7 | 37.1 ± 3.3 | | | 34.9 ± 0.7 | | 45.0 ± 0.8 | | 48.1 ± 4.4 | | 43.8 ± 3.1 | | 42.5 ± 1.8 | | 48.1 ± 1.2 | | 32.4 ± 0.1 | 34 ± 5.0 | | 42.8 ± 2.7 | |
| Y (89) | 7330 ± 1855 | 5670 ± 1137 | 5470 ± 142 | | | 6500 ± 135 | | 7260 ± 32 | | 7300 ± 726 | | 8440 ± 837 | | 7170 ± 420 | | 6700 ± 80 | | 7130 ± 260 | 5240 ± 985 | | 6960 ± 493 | |
| Zr (90) | 0.32 ± 0.06 | 0.31 ± 0.04 | 0.30 ± 0.01 | | | 0.31 ± 0.01 | | 0.31 ± 0.05 | | 0.31 ± 0.01 | | 0.32 ± 0.03 | | 0.30 ± 0.04 | | 0.29 ± 0.01 | | 0.3 ± 0.03 | 0.29 ± 0.03 | | 0.28 ± 0.02 | |
| Nb (93) | 0.04 ± 0.00 | 0.04 ± 0.01 | 0.04 ± 0.01 | | | 0.04 ± 0.00 | | 0.04 ± 0.01 | | 0.04 ± 0.01 | | 0.04 ± 0.01 | | 0.04 ± 0.01 | | 0.04 ± 0.01 | | 0.04 ± 0.01 | 0.04 ± 0.01 | | 0.05 ± 0.01 | |
| Sn (118) | 0.04 ± 0.02 | 0.02 ± 0.01 | 0.03 ± 0.02 | | | 0.03 ± 0.01 | | 0.03 ± 0.01 | | 0.04 ± 0.02 | | 0.02 ± 0.04 | | 0.03 ± 0.02 | | 0.02 ± 0.03 | | 0.02 ± 0.06 | 0.04 ± 0.03 | | 0.02 ± 0.01 | |
| Cs (133) | n.d. | n.d. | n.d. | | | 0.01 ± 0.03 | | n.d. | | n.d. | | n.d. | | n.d. | | n.d. | | n.d. | 0.02 ± 0.03 | | n.d. | |

| Ba (137) | 0.04 ± 0.01 | 0.04 ± 0.02 | 0.06 ± 0.07 | 0.04 ± 0.01 | 0.04 ± 0.00 | 0.03 ± 0.01 | 0.03 ± 0.02 | 0.02 ± 0.05 | 0.03 ± 0.01 | 0.02 ± 0.05 | 0.02 ± 0.03 | 0.03 ± 0.00 |
| --- | --- | --- | --- | --- | --- | --- | --- | --- | --- | --- | --- | --- |
| La (139) | 141200 ± 4050 | 132900 ± 2000 | 132100 ± 6100 | 137000 ± 9690 | 135300 ± 12220 | 131900 ± 13990 | 128500 ± 8070 | 130700 ± 9530 | 134000 ± 2460 | 128000 ± 10160 | 130900 ± 6000 | 127700 ± 1080 |
| Ce (140) | 248000 ± 7390 | 249000 ± 6390 | 245000 ± 14420 | 251000 ± 16240 | 248000 ± 18990 | 243000 ± 17480 | 239000 ± 13890 | 241000 ± 16300 | 243000 ± 5530 | 240000 ± 17870 | 242000 ± 8190 | 236000 ± 5220 |
| Pr (141) | 26900 ± 532 | 28000 ± 749 | 28100 ± 1506 | 27900 ± 1799 | 27700 ± 2300 | 27100 ± 3000 | 27400 ± 1530 | 27300 ± 1683 | 26900 ± 650 | 27700 ± 1288 | 27100 ± 1376 | 27600 ± 1069 |
| Nd (146) | 91100 ± 4200 | 99200 ± 1701 | 102600 ± 6940 | 100300 ± 6660 | 98900 ± 7220 | 97300 ± 9690 | 100100 ± 5210 | 97800 ± 7760 | 97800 ± 2690 | 102300 ± 6010 | 98500 ± 4440 | 103700 ± 4630 |
| Sm (147) | 11440 ± 1112 | 12470 ± 995 | 12920 ± 943 | 13380 ± 783 | 13570 ± 1264 | 13370 ± 1491 | 13790 ± 806 | 13140 ± 980 | 12920 ± 183 | 13900 ± 472 | 12520 ± 593 | 14490 ± 708 |
| Eu (153) | 1183 ± 33 | 1196 ± 88 | 1181 ± 49 | 1254 ± 59 | 1359 ± 159 | 1304 ± 124 | 1239 ± 98 | 1202 ± 76 | 1193 ± 29 | 1212 ± 35 | 1096 ± 73 | 1318 ± 68 |
| Gd (157) | 7960 ± 1393 | 8260 ± 1118 | 8530 ± 935 | 9060 ± 455 | 9250 ± 960 | 9110 ± 1111 | 9180 ± 687 | 8690 ± 387 | 8520 ± 172 | 8850 ± 353 | 7900 ± 490 | 9910 ± 350 |
| Tb (159) | 618 ± 117 | 594 ± 107 | 597 ± 47 | 667 ± 26 | 702 ± 75 | 701 ± 99 | 721 ± 62 | 670 ± 33 | 646 ± 12 | 702 ± 10 | 593 ± 58 | 765 ± 21 |
| Dy (163) | 2010 ± 435 | 1740 ± 401 | 1724 ± 128 | 2040 ± 78 | 2190 ± 193 | 2200 ± 331 | 2330 ± 231 | 2120 ± 71 | 2000 ± 35 | 2340 ± 14 | 1860 ± 304 | 2470 ± 160 |
| Ho (165) | 245 ± 47 | 194 ± 45 | 190 ± 18 | 225 ± 8 | 247 ± 22 | 250 ± 37 | 269 ± 31 | 242 ± 6 | 226 ± 7 | 278 ± 8 | 207 ± 48 | 282 ± 25 |
| Er (166) | 475 ± 67 | 370 ± 59 | 360 ± 27 | 394 ± 16 | 431 ± 39 | 432 ± 63 | 469 ± 50 | 418 ± 6 | 394 ± 16 | 502 ± 13 | 382 ± 70 | 484 ± 45 |
| Tm (169) | 24.3 ± 2.9 | 15.0 ± 3.4 | 14.0 ± 1.1 | 16.2 ± 0.5 | 19.1 ± 1.7 | 19.4 ± 3.1 | 22.2 ± 2.9 | 18.9 ± 0.9 | 17 ± 0.8 | 25.9 ± 1.4 | 17.2 ± 6.5 | 21.8 ± 2.3 |
| Yb (172) | 101 ± 13.4 | 64.2 ± 13.7 | 59.3 ± 7 | 67.7 ± 0.9 | 78.6 ± 5.9 | 78.5 ± 11.6 | 88.1 ± 10.8 | 74.7 ± 3.6 | 67.3 ± 3.0 | 104 ± 4.9 | 68.7 ± 25.4 | 86.5 ± 7.5 |
| Lu (175) | 9.68 ± 1.54 | 6.30 ± 1.26 | 5.87 ± 0.66 | 6.68 ± 0.16 | 7.79 ± 0.57 | 7.54 ± 1.21 | 8.28 ± 0.98 | 7.07 ± 0.18 | 6.42 ± 0.33 | 9.82 ± 0.34 | 6.66 ± 2.35 | 8.45 ± 0.80 |
| Hf (178) | 8.97 ± 2.09 | 7.57 ± 1.68 | 7.39 ± 0.49 | 8.29 ± 0.38 | 9.09 ± 0.47 | 8.88 ± 1.23 | 9.07 ± 0.92 | 8.13 ± 0.27 | 7.7 ± 0.29 | 8.61 ± 0.04 | 7.04 ± 0.94 | 9.35 ± 0.91 |
| Ta (181) | 1.29 ± 0.25 | 1.01 ± 0.17 | 0.94 ± 0.04 | 1.06 ± 0.03 | 1.19 ± 0.04 | 1.18 ± 0.15 | 1.24 ± 0.16 | 1.07 ± 0.01 | 1.02 ± 0.06 | 1.22 ± 0.03 | 0.94 ± 0.19 | 1.23 ± 0.12 |
| Pb (208) | 6.29 ± 7.34 | 18.6 ± 26.6 | 32.7 ± 14.9 | 23.5 ± 3.7 | 25.1 ± 0.4 | 42.6 ± 5.3 | 35.1 ± 5.7 | 38.7 ± 2.1 | 58 ± 4.3 | 10.1 ± 1.4 | 34.7 ± 5.1 | 48.3 ± 6.7 |
| Th (232) | 1357 ± 1579 | 4050 ± 5890 | 7280 ± 3110 | 5860 ± 997 | 6170 ± 115 | 10580 ± 1367 | 7930 ± 1235 | 8740 ± 420 | 13090 ± 720 | 2490 ± 325 | 8470 ± 1385 | 13730 ± 948 |
| U (238) | 25.0 ± 0.4 | 18.9 ± 4.9 | 19.2 ± 1.3 | 25.8 ± 0.5 | 29.2 ± 2.9 | 30.1 ± 3.3 | 30.1 ± 2.1 | 26.0 ± 1.0 | 25.5 ± 1.6 | 33.5 ± 1.1 | 24.3 ± 5.0 | 29.5 ± 0.6 |

^a^ The isotope measured is quoted in brackets

^b^ Errors are quoted at the 2σ level. n.d. = not detected

**Table S3** Fractional atomic coordinates and isotropic displacement parameters of parisite–(Ce) at ambient conditions

| Atom  name | Atom type | Wyckoff  position | Site occupation | x/a | y/b | z/c | U_iso_  (or equivalent) |
| --- | --- | --- | --- | --- | --- | --- | --- |
| Ce01a | Ce | 4a | 0.885(6) | 0.47815(6) | 0.241664(6) | 0.163315(2) | 0.0082(2) |
| Ce01b | Ca | 4a | 0.115(6) | cf. pos. Ce01a | cf. pos. Ce01a | cf. pos. Ce01a | cf. pos. Ce01a |
| Ce02a | Ce | 4a | 0.884(7) | 0.80633(7) | 0.25142(7) | 0.16309(2) | 0.0063(2) |
| Ce02b | Ca | 4a | 0.116(7) | cf. pos. Ce02a | cf. pos. Ce02a | cf. pos. Ce02a | cf. pos. Ce02a |
| Ce03a | Ce | 4a | 0.894(7) | 0.14609(8) | 0.25472(6) | 0.16352(2) | 0.0089(2) |
| Ce03b | Ca | 4a | 0.106(7) | cf. pos. Ce03a | cf. pos. Ce03a | cf. pos. Ce03a | cf. pos. Ce03a |
| Ce04a | Ce | 4a | 0.861(7) | 0.53534(5) | 0.24288(6) | 0.33583(2) | 0.0078(2) |
| Ce04b | Ca | 4a | 0.139(7) | cf. pos. Ce04a | cf. pos. Ce04a | cf. pos. Ce04a | cf. pos. Ce04a |
| Ce05a | Ce | 4a | 0.846(8) | 0.20384(9) | 0.25443(6) | 0.33605(2) | 0.0076(2) |
| Ce05b | Ca | 4a | 0.154(8) | cf. pos. Ce05a | cf. pos. Ce05a | cf. pos. Ce05a | cf. pos. Ce05a |
| Ce06a | Ce | 4a | 0.857(8) | 0.86314(7) | 0.25067(7) | 0.33568(2) | 0.0058(2) |
| Ce06b | Ca | 4a | 0.143(8) | cf. pos. Ce06a | cf. pos. Ce06a | cf. pos. Ce06a | cf. pos. Ce06a |
| Ca01a | Ca | 4a | 0.867(4) | 0.0928(3) | 0.25254(10) | -0.00165(14) | 0.0091(4) |
| Ca01b | Nd | 4a | 0.133(4) | cf. pos. Ca01a | cf. pos. Ca01a | cf. pos. Ca01a | cf. pos. Ca01a |
| Ca02a | Ca | 4a | 0.914(3) | 0.9215(2) | 0.25454(18) | 0.49856(10) | 0.0111(3) |
| Ca02b | Nd | 4a | 0.086(3) | cf. pos. Ca02a | cf. pos. Ca02a | cf. pos. Ca02a | cf. pos. Ca02a |
| Ca03a | Ca | 4a | 0.892(4) | 0.7532(3) | 0.23659(16) | -0.00182(15) | 0.0075(3) |
| Ca03b | Nd | 4a | 0.108(4) | cf. pos. Ca03a | cf. pos. Ca03a | cf. pos. Ca03a | cf. pos. Ca03a |
| F01 | F | 4a |  | 0.9778(5) | 0.0821(4) | 0.16029(16) | 0.0121(5) |
| F02 | F | 4a |  | 0.3060(4) | 0.0802(5) | 0.14485(15) | 0.0118(6) |
| F03 | F | 4a |  | 0.6503(4) | 0.0852(5) | 0.17883(15) | 0.0112(6) |
| F04 | F | 4a |  | 0.0367(5) | 0.0812(4) | 0.33636(16) | 0.0144(6) |
| F05 | F | 4a |  | 0.6958(4) | 0.0822(5) | 0.31803(15) | 0.0105(5) |
| F06 | F | 4a |  | 0.3736(4) | 0.0829(5) | 0.35230(14) | 0.0116(6) |
| O01 | O | 4a |  | 0.3979(5) | 0.3178(6) | 0.07826(19) | 0.0171(9) |
| O02 | O | 4a |  | 0.2500(5) | 0.4295(6) | 0.03424(18) | 0.0116(8) |
| O03 | O | 4a |  | 0.2681(5) | 0.4505(6) | 0.11359(17) | 0.0120(8) |
| O04 | O | 4a |  | 0.1102(5) | 0.1346(5) | 0.07784(18) | 0.0130(7) |
| O05 | O | 4a |  | 0.6014(6) | 0.3868(6) | 0.03011(18) | 0.0146(8) |
| O06 | O | 4a |  | 0.6283(5) | 0.3507(6) | 0.11034(18) | 0.0134(8) |
| O07 | O | 4a |  | 0.8449(5) | 0.2957(7) | 0.07745(18) | 0.0153(8) |
| O08 | O | 4a |  | 0.9964(5) | 0.4474(6) | 0.11020(18) | 0.0136(8) |
| O09 | O | 4a |  | 0.9483(5) | 0.4340(7) | 0.03060(18) | 0.0162(9) |
| O10 | O | 4a |  | 0.1124(5) | 0.2933(6) | 0.24861(18) | 0.0125(7) |
| O11 | O | 4a |  | 0.9697(5) | 0.4306(6) | 0.20759(16) | 0.0110(7) |
| O12 | O | 4a |  | 0.9912(5) | 0.4377(6) | 0.28738(17) | 0.0115(7) |
| O13 | O | 4a |  | 0.8485(5) | 0.1337(7) | 0.24833(16) | 0.0135(9) |
| O14 | O | 4a |  | 0.3582(5) | 0.3657(6) | 0.28932(18) | 0.0121(8) |
| O15 | O | 4a |  | 0.3323(5) | 0.3606(6) | 0.20967(18) | 0.0126(8) |
| O16 | O | 4a |  | 0.5607(5) | 0.3177(6) | 0.24862(18) | 0.0143(8) |
| O17 | O | 4a |  | 0.7081(5) | 0.4488(6) | 0.28879(16) | 0.0113(7) |
| O18 | O | 4a |  | 0.6843(5) | 0.4484(6) | 0.20887(17) | 0.0120(8) |
| O19 | O | 4a |  | 0.9571(5) | 0.2948(6) | 0.41840(17) | 0.0142(8) |
| O20 | O | 4a |  | 0.0842(5) | 0.4430(6) | 0.38382(17) | 0.0118(7) |
| O21 | O | 4a |  | 0.0947(5) | 0.4371(6) | 0.46343(19) | 0.0140(8) |
| O22 | O | 4a |  | 0.5092(5) | 0.3155(6) | 0.41946(17) | 0.0138(8) |
| O23 | O | 4a |  | 0.3530(4) | 0.4504(5) | 0.38793(16) | 0.0107(7) |
| O24 | O | 4a |  | 0.4001(5) | 0.4321(6) | 0.46736(18) | 0.0139(8) |
| O25 | O | 4a |  | 0.7177(5) | 0.3496(6) | 0.38672(18) | 0.0145(8) |
| O26 | O | 4a |  | 0.2229(5) | 0.1344(5) | 0.41943(18) | 0.0128(7) |
| O27 | O | 4a |  | 0.7467(5) | 0.3843(6) | 0.46594(18) | 0.0141(8) |
| C01 | C | 4a |  | 0.3058(6) | 0.3987(7) | 0.07561(19) | 0.0069(8) |
| C02 | C | 4a |  | 0.6149(7) | 0.4553(7) | 0.0732(2) | 0.0099(8) |
| C03 | C | 4a |  | 0.9302(6) | 0.3904(7) | 0.07308(20) | 0.0085(8) |
| C04 | C | 4a |  | 0.0246(5) | 0.3886(7) | 0.24815(19) | 0.0066(8) |
| C05 | C | 4a |  | 0.3487(6) | 0.4520(9) | 0.24911(19) | 0.0080(9) |
| C06 | C | 4a |  | 0.6516(6) | 0.4036(8) | 0.2484(2) | 0.0076(8) |
| C07 | C | 4a |  | 0.0473(6) | 0.3914(7) | 0.42143(19) | 0.0074(8) |
| C08 | C | 4a |  | 0.4200(5) | 0.3991(7) | 0.42346(18) | 0.0059(8) |
| C09 | C | 4a |  | 0.7318(6) | 0.4566(6) | 0.42351(18) | 0.0064(8) |


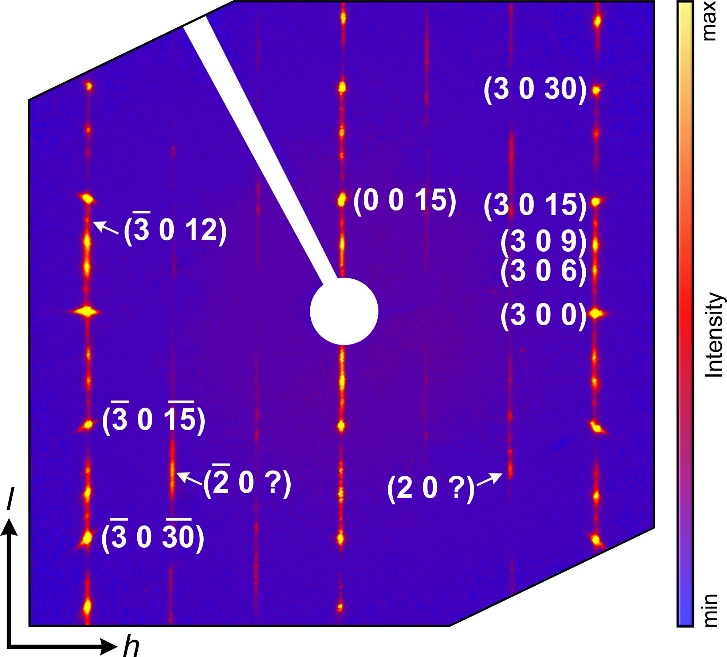


**Fig. S1** Reciprocal space map of röntgenite–(Ce) (calculated from results obtained from crystal #3)
