# Supplementary figures and images for: The parisite–(Ce) enigma: challenges in the identification of fluorcarbonate minerals
Source: Mineral Petrol. 2020 Oct 10;115(1):1–19. doi: 10.1007/s00710-020-00723-x (PMC7831744; doi:10.1007/s00710-020-00723-x)

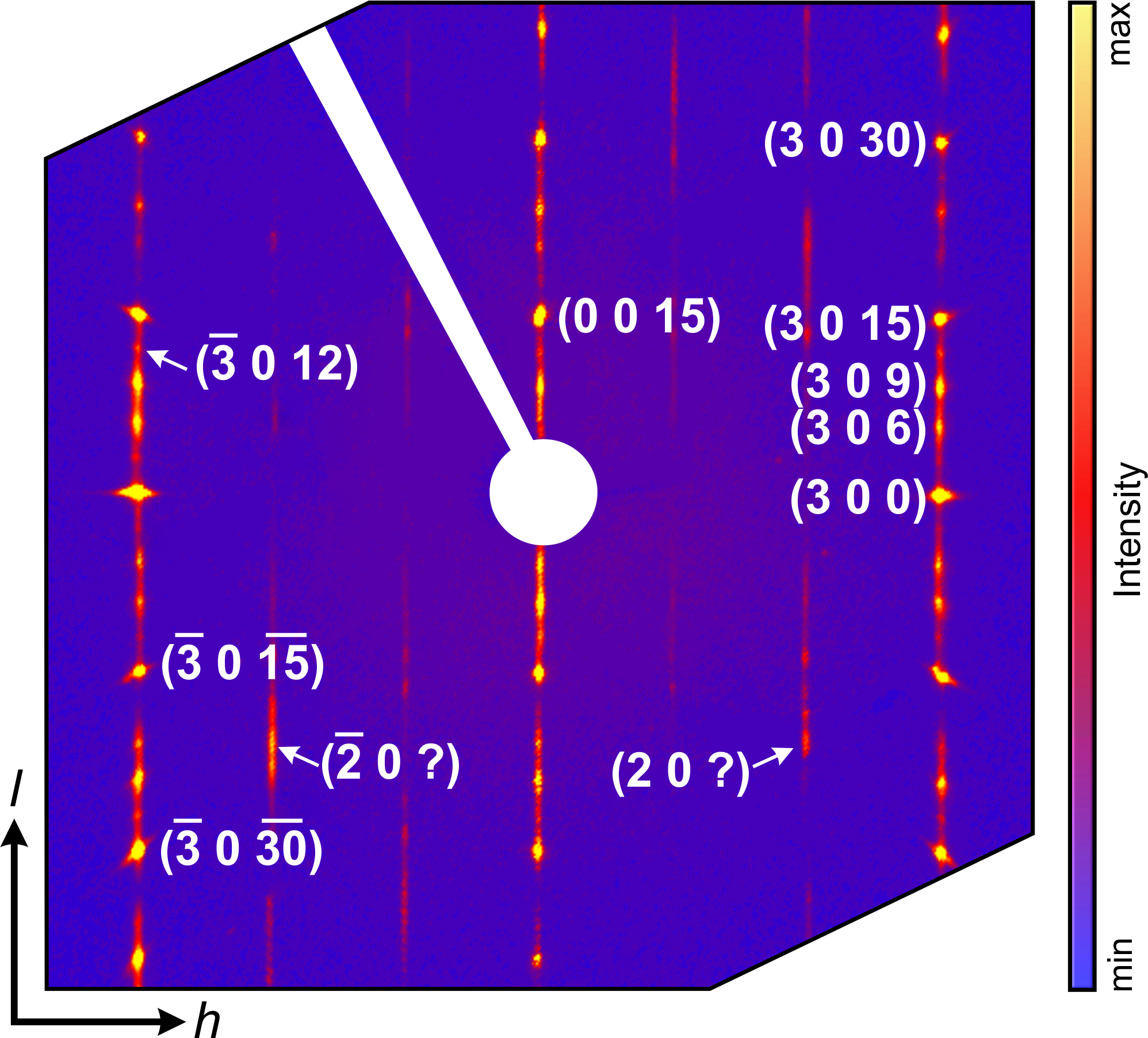

Supplement: Supplementary file 2 — (TIF 14084 kb) [file 710_2020_723_MOESM2_ESM.tif]

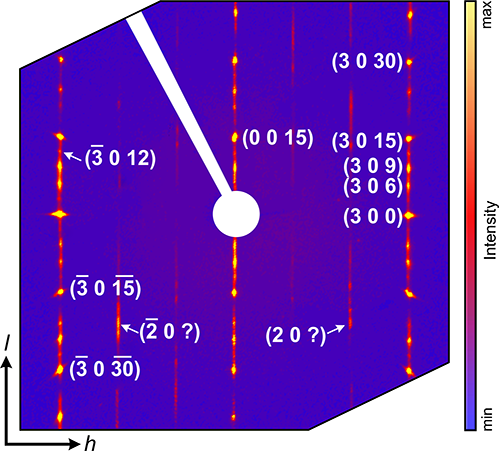

Supplement: Supplementary file 3 — High Resolution image (PNG 662 kb) [file 710_2020_723_Fig14_ESM.png]
